# The Human Remains From the MIS 6 Site of Grotta Del Poggio (Cilento, Southern Italy): A Taxonomic and Chronological Reassessment

**DOI:** 10.1002/ajpa.70188

**Published:** 2025-12-28

**Authors:** Erica Piccirilli, Rita Sorrentino, Francesca Seghi, Antonino Vazzana, Maria Giovanna Belcastro, Sahra Talamo, Katerina Harvati, Matteo Bettuzzi, Maria Pia Morigi, Gerhard Weber, Giulia Capecchi, Vincenzo Spagnolo, Ivan Martini, Adriana Moroni, Francesco Boschin, Stefano Ricci, Stefano Benazzi

**Affiliations:** ^1^ Department of Cultural Heritage University of Bologna Bologna Italy; ^2^ Department of Biological, Geological and Environmental Sciences University of Bologna Bologna Italy; ^3^ Department of Chemistry G. Ciamician University of Bologna Bologna Italy; ^4^ Paleoanthropology, Senckenberg Centre for Human Evolution and Palaeoenvironment Eberhard Karls University of Tübingen Tübingen Germany; ^5^ Department of Geosciences, Institute for Archaeological Sciences Eberhard Karls University of Tübingen Tübingen Germany; ^6^ DFG Centre of Advanced Studies ‘Words, Bones, Genes, Tools’ Eberhard Karls University of Tübingen Tübingen Germany; ^7^ Department of Physics and Astronomy “Augusto Righi” University of Bologna Bologna Italy; ^8^ Department of Evolutionary Anthropology University of Vienna Vienna Austria; ^9^ Human Evolution and Archaeological Science (HEAS) University of Vienna Vienna Austria; ^10^ Department of Physical Sciences, Earth and Environment University of Siena Siena Italy; ^11^ Department of Physical Sciences, Earth and Environment, Research Unit of Prehistory and Anthropology University of Siena Siena Italy; ^12^ Centro Studi Sul Quaternario (CeSQ ODV) Arezzo Italy; ^13^ Istituto Italiano di Paleontologia Umana (IsIPU) Frosinone Italy

**Keywords:** AMS radiocarbon dating, geometric morphometrics, human remains, Neanderthal, recent *Homo sapiens*

## Abstract

**Objectives:**

Grotta del Poggio is a key site for exploring the Middle Paleolithic in southern Italy, as it contains a pivotal anthropogenic deposit, mainly attributed to MIS 6, while in the Metal Ages, the cavity was used as a burial place. Excavations in the cave's deposit led to the discovery of a human molar and a human talus. A preliminary morphological evaluation of the talus concluded that it belonged to *Homo sapiens* . Conversely, the molar exhibited Neanderthal‐like morphology. Here, we perform a taxonomic and chronological reassessment of these human remains.

**Materials and Methods:**

The molar's crown and root morphology were examined and analyzed using linear measurements, 2D geometric morphometrics (GM) of the crown outline, and 3D GM of the enamel‐dentin junction (EDJ) and cemento‐enamel junction (CEJ). The talus was investigated through a 3D GM analysis of its whole shape. Both specimens were compared with *Homo neanderthalensis* and *H. sapiens* samples. Moreover, we performed radiocarbon dating on the talus to elucidate its absolute age.

**Results:**

The molar's non‐metric traits, linear measurements, crown outline, EDJ, and CEJ confirmed its Neanderthal attribution, while the talus was attributed to recent *H. sapiens* . Radiocarbon dating ascribed the talus to the Middle Bronze age.

**Discussion:**

This study clarifies the taxonomic attribution of the two already known human remains from Grotta del Poggio, revising one of the oldest Neanderthal remains in Italy based on cutting‐edge methodologies, and elucidating the reasons why a morphologically modern talus was recovered during the excavation of the Mousterian deposit.

## 1 Introduction

The presence of *H. neanderthalensis* in southern Italy has long been documented thanks to a number of human remains, mostly found in association with Mousterian lithic and faunal material in the regions of Campania, Calabria and Apulia (Cardini 1955; A. C. Blanc 1961a, 1961b; di Palma Cesnola and Messeri 1967; Vigliardi 1968; von Borzatti Löwenstern 1969; Ascenzi and Segre 1971; Messeri 1975; Messeri and di Palma Cesnola 1976; Borgognini Tarli 1983; Bonfiglio et al. 1986; Mallegni et al. 1987; Mallegni and Ronchitelli 1989; Pesce Delfino and Vacca 1993; Mallegni and Trinkaus 1997; Bologna et al. 1994; Benazzi, Douka, et al. 2011; Benazzi, Viola, et al. 2011; Benazzi et al. 2013; Fabbri et al. 2016; Moroni et al. 2018; Fabbri and Vincenti 2020; Oxilia et al. 2022; Seghi et al. 2024). Notably, the discovery of bones and teeth in secure stratigraphic positions provides direct evidence for the presence of the Neanderthals in these areas from at least the Marine Isotopic Stage (MIS) 6 (200–130 ka; Boscato et al. 2009) and up to ~43 ka cal BP (Higham et al. 2024), considerably predating and later coinciding with the establishment of *H. sapiens* in Italy around 44–43 ka BP (Benazzi, Douka, et al. 2011; Higham et al. 2024). These findings therefore indicate a relatively brief period of chronological overlap with very limited, if any, cohabitation and cultural/biological interaction between the two human groups, contrary to what is believed to have occurred in other regions of Europe (Sümer et al. 2024).

Until recently, Europe from before ca. 45 ka was considered exclusively dominated by Neanderthals and Mousterian lithic industries were attributed to this taxon, even when they were not found in association with human remains. However, recent studies based on new discoveries and the re‐examination of old collections suggest that incursions of *H. sapiens* into southern Europe (Harvati et al. 2019; Slimak et al. 2022) may have taken place even earlier than previously suspected, at times when the material culture of these *H. sapiens* populations was in most cases virtually indistinguishable from that of Neanderthals.

**FIGURE 1:**
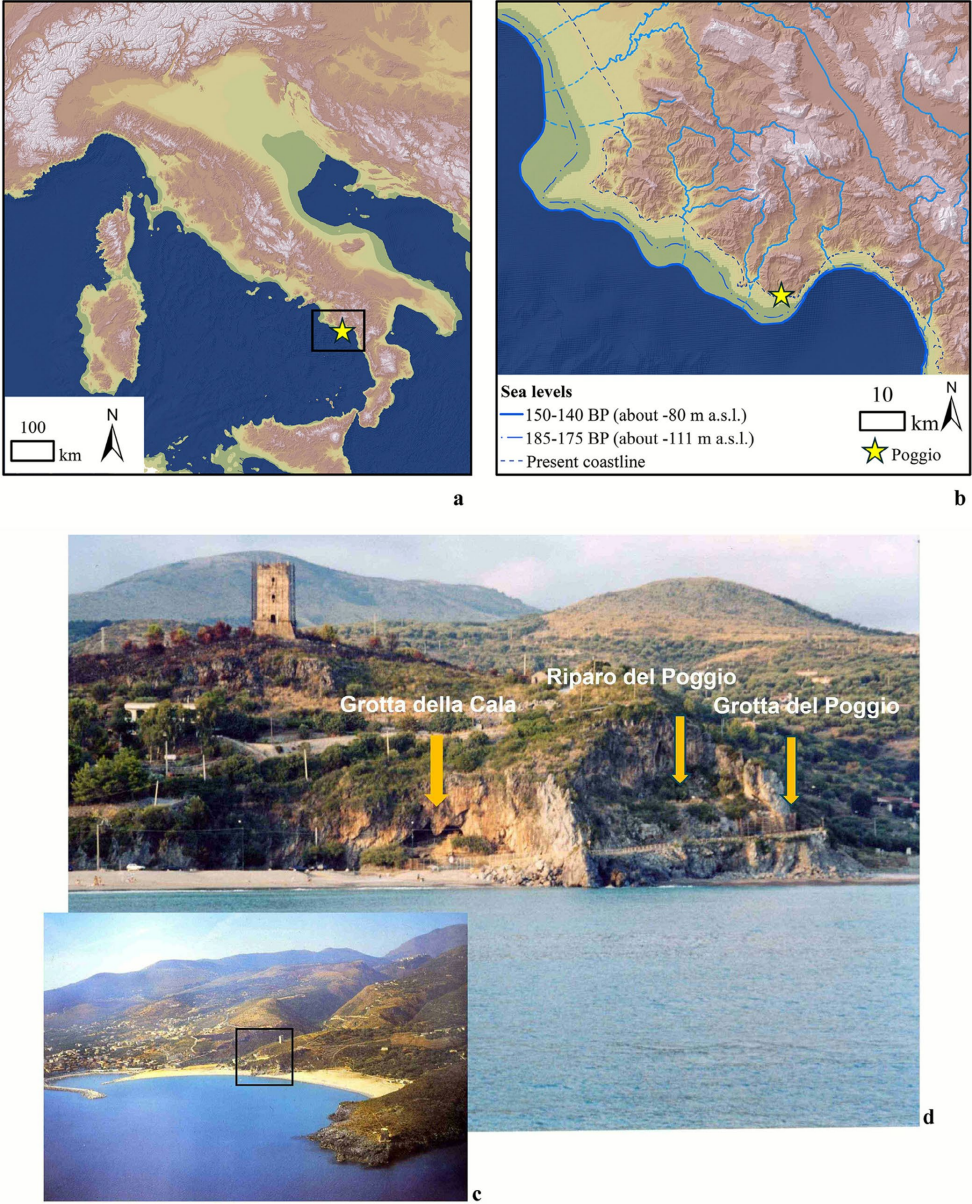
(a, b) Palaeogeographic maps of Italy during MIS 6 with the location of the Poggio complex highlighted by the yellow star; (c) location of Poggio with respect to the village of Marina di Camerota; (d) view of the Poggio complex from the sea.

According to this multifaceted scenario, the site of Poggio, located east of Marina di Camerota in Campania (Figure 1a–d), can be considered an ideal location for testing the new hypotheses, due to the presence of human remains and to its impressive stratigraphic sequence, more than 20 m thick, extending chronologically from MIS 7 (~250–190 ka BP) to the Metal Ages (Gambassini and Ronchitelli 1998; Boscato et al. 2009). In this site, which comprises a cave (Grotta del Poggio) and a rock‐shelter (Riparo del Poggio), most of the anthropogenic deposits represent Mousterian occupations (14 m in thickness, including both the shelter and the cave). These include several layers encompassing MIS 6 pre‐Levallois assemblages (8.5 m in thickness), allowing us to zoom in on this little‐known phase of the Italian Middle Paleolithic.

During the excavations in the Middle Paleolithic deposit of the cave (about 6 m thick), which is almost entirely attributed to MIS 6, a human molar was found in 1966 (Palma di Cesnola's field notes, 7th May 1966), while two other human remains, a talus and a femoral head, initially both identified as human, were recovered in 1964 and 1974, respectively. The three finds were described by Messeri and di Palma Cesnola (1976), who noted clear Neanderthal features on the tooth, such as massive roots and the relative position of the cusps, and modern human features on the talus and femoral head. However, a subsequent re‐evaluation of the femoral head led to the conclusion that it was not human but faunal (di Palma Cesnola and Messeri 1967; Messeri 1975; Messeri and di Palma Cesnola 1976; Orban 1988; di Palma Cesnola 1996; Alciati et al. 2005), and identified as cervid (AM's personal communication to EP). Metal age human remains, among them a small portion of a rib, were found in 1974 during the restoration of the cut profile in the excavation trench.

Given its geochronological and biostratigraphic context, the tooth may represent one of the most ancient Neanderthal remains as yet known for the Italian territory (Alciati et al. 2005; Buzi et al. 2021). Although human remains from Grotta del Poggio have long been known to the scientific community (di Palma Cesnola 2001; Alciati et al. 2005; Buzi et al. 2021; Janković et al. 2024), a detailed morphological and morphometric analysis aided by 3D digital methods is still lacking. The present article aims to fill this gap, providing a reassessment of the taxonomy of the tooth and talus on the basis of their geometric morphometric evaluation. Additionally, a radiocarbon date is provided for the talus to improve the understanding of its chronology and context, as well as for the rib fragment to provide a comparison for the date of the talus.

### 1.1 History of Research and Site Presentation

Grotta and Riparo del Poggio are part of a system of caves and shelters opening into the rock wall of the Poggio limestone promontory, located 500 m east of Marina di Camerota (Salerno, southern Italy; Figure 1). Grotta del Poggio, located ca. 10 masl, is presently a small cave, measuring 6 m in length and 5 m in width. It was investigated and described for the first time by P. Parenzan in the 1950s (Parenzan 1954, 1956; Chiappella 1956), when almost half of the cave, originally 15 m long, had not yet been destroyed during the construction of the coastal road by the Municipality of Camerota in 1957–1958. In 1964, research at the site was resumed by A. Palma di Cesnola of the University of Siena in collaboration with P. Gambassini of the same university and went on, with some interruptions, until 1974. During this period, the two scholars realized that the shelter and cave were part of a vast interconnected complex, as some of the cave's layers outcropping in the cut of the road were in clear continuity with the sediments of the adjacent shelter (Figure S1; Palma di Cesnola's field notes; Boscato et al. 2009). Based on these observations, as well as on the present conformation of the shelter, P. Gambassini and A. Palma di Cesnola were able to reconstruct the articulated formation process that gave rise to the morphology of Poggio as it is today. According to the model proposed by P. Gambassini, the promontory was originally traversed by a large karstic cave that developed along a fault plane in an east–west direction, parallel to the cliff. To the east, this cave considerably narrowed into a tunnel‐shaped cavity, known today as Grotta del Poggio. The seaward wall and vault of the original cave were gradually dismantled by sea erosion during the high‐stand of MIS 7, documented at the base of the continental deposit (see below). The collapse of the vault continued afterwards, leaving traces in the huge limestone blocks that are still present in the stratigraphy (Gambassini and Ronchitelli 1998; Boscato et al. 2009; Caramia 2008). Due to the importance of the site, scientific interest in the Grotta del Poggio has recently been revived. In 2019, the whole series of the cave was the subject of new sedimentological analyses, and a Uranium‐Thorium (U‐Th) and Electron spin resonance (ESR) dating programme was started, followed by the zooarchaeological study. Finally, in 2022, a consortium of universities (Siena, Bologna and Tübingen) has resumed excavations in the cave with the aim of going through the whole series again to obtain more information using modern excavation techniques. This work is carried out in the framework of the ERC Project FIRSTSTEPS, which aims to apply an interdisciplinary approach to investigate the early dispersals and contacts of *H. sapiens* in south‐eastern Europe and Italy, a key area of sustained interaction during the Middle and Late Pleistocene. Although preliminary, the results of these studies (article in preparation) and of the new excavations confirm the chronological and palaeoenvironmental model proposed by Gambassini (Gambassini and Ronchitelli 1998; Boscato et al. 2009), as well as the integrity of the cave stratigraphic succession investigated in the 20th century.

The whole sequence (cave and shelter, a 23 m‐thick deposit in total, including 17 m of archaeological levels) lies on a strongly cemented marine conglomerate (layer 23 of the shelter located at 9.5 masl) composed of large, rounded limestone pebbles, perforated by lithophages, with a matrix of grayish‐colored sand (Figure S1). This conglomerate (termed by Gambassini “Poggio Marine Unit”—UMP) has been attributed to MIS 7 and is one of the key elements in the correlation between different coastal deposits as it outcrops at several points on the Cilento coastline (e.g., Riparo di Santa Caterina, Grotta degli Infreschi, Grotta Grande and Riparo del Molare; Gambassini and Ronchitelli 1998).

As mentioned above, the Middle Paleolithic deposit of the cave can be almost entirely ascribed to MIS 6 on both geochronological and biostratigraphic bases (Sala 1979; Boscato et al. 2009), and represents, together with layer 18 of the shelter, the oldest portion of the Poggio succession (Figure S1). Thirteen layers (14–2 in stratigraphic order) have been identified in the continental deposit overlying the marine conglomerate, all of which contain lithics and faunal remains, except for the sterile breccias of layer 14. The sedimentation of layers 14 to 3 is relatively homogeneous, characterized by brown sediments, alternating with breccias rich in stones and, underneath, lenses of dark sand. In particular, layer 6, where the tooth was discovered, was originally described as “brown sediment with medium to fine debris, little clay and few concretions, with large amounts of faunal remains, on average 30 cm thick” (Palma di Cesnola's field notes translated by AM; Bartolomei et al. 1976; Caramia 2008). Conversely, there is a clear cultural and sedimentological discontinuity between layers 13–3 and 2, the latter consisting of yellowish‐brown soil containing large limestone blocks. In contrast to layer 2, which looks like a thick palimpsest, layers 10 to 3 have yielded horizontally arranged short‐term palimpsests with fireplaces (Palma di Cesnola's field notes). Above layer 2, there are remnants of deposits with Late Mousterian (layer 1) and Upper Paleolithic materials, which are the result of erosive phenomena that affected the Poggio from the end of the Mousterian cycle (Figure S1). The last occupation of the cave dates back to the Holocene, when it was used as a burial place as evidenced by the occurrence of pottery shards and human remains of *H. sapiens* individuals from the Metal Ages (Chiappella 1956; Messeri and di Palma Cesnola 1976; Sala 1979; Gambassini and Ronchitelli 1998; Caramia 2008; Boscato et al. 2009).

**FIGURE 2:**
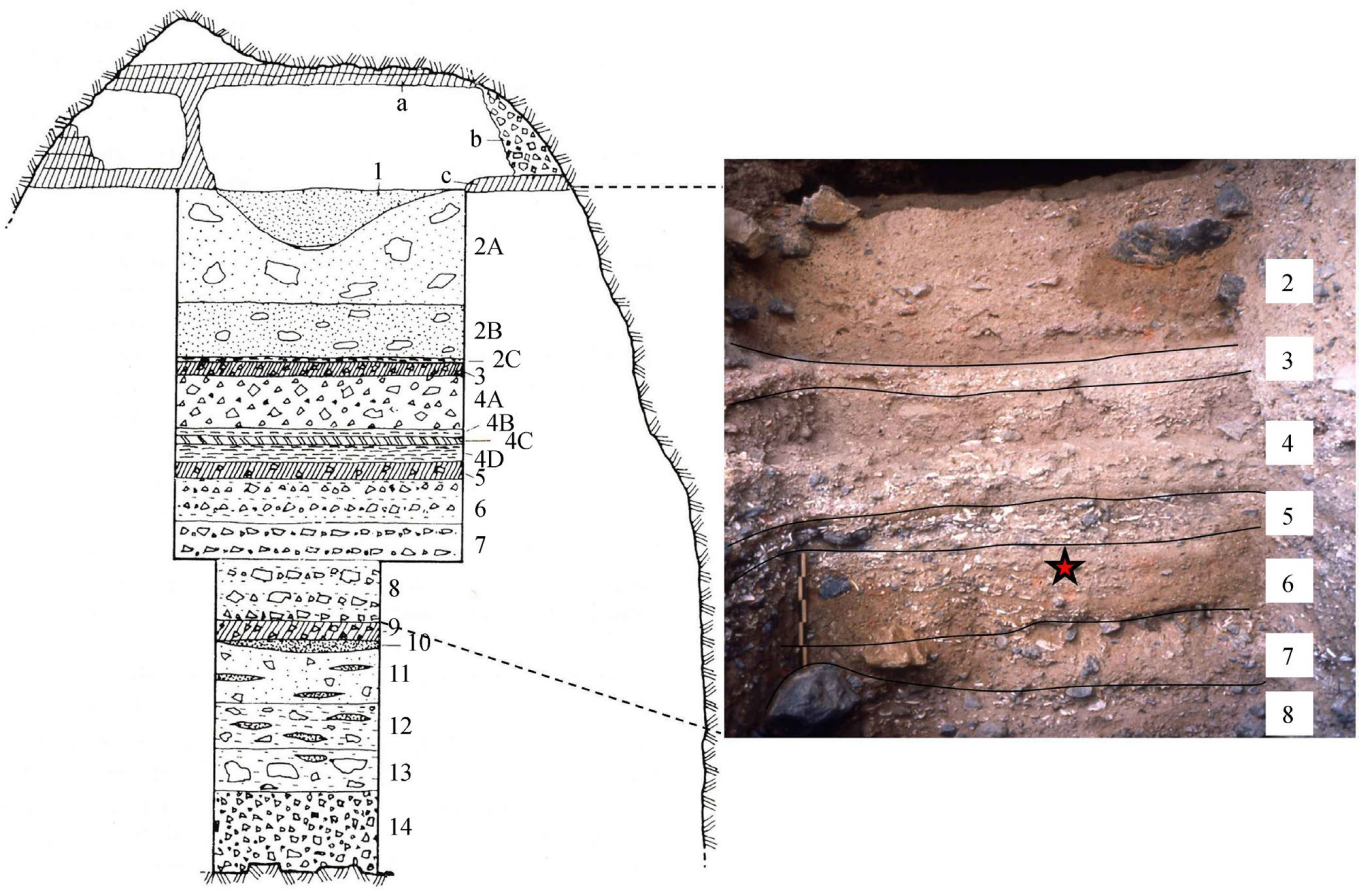
Poggio. Profile of the cave and of the stratigraphic sequence brought to light in the ‘60s (on the left—after Palma di Cesnola 1969); stratigraphic detail of layers 2 to 8, with the position of the Neanderthal tooth indicated by the red star (on the right—from Palma di Cesnola's archive modified).

Layers 14–2 of the cave correspond to layers 19–18 of the shelter (Figures 2 and S1). More specifically, layers 10–3 can be chronologically correlated with layer 18‐sub‐layers L‐H of the shelter, and layer 2 is in phase with layer 18‐sub‐layers G‐C of the shelter (Bartolomei et al. 1976; Boscato et al. 2009). This whole package (with a total thickness of ca. 9 m, of which about 5 m are also in the cave) contains pre‐Levallois lithic assemblages and is sealed in the shelter by a stalagmitic crust tentatively dated to MIS 5e as the overlying layer 17 has yielded a Thermoluminescence (TL) date of 111.800 ± 9.500 corresponding to MIS 5d (Figure S1; Boscato et al. 2009), which represents a *terminus ante quem* for the Middle Paleolithic deposit of the cave and layer 18 of the shelter. From layer 17 onwards, the Levallois production begins, chronologically coinciding with the onset of the Levallois in the region (Aureli and Ronchitelli 2018; Spagnolo et al. 2020; Carmignani et al. 2021), as also attested in the nearby sites of Riparo di Santa Caterina and Grotta degli Infreschi, where the first layers with Levallois were dated to 111.300 + 10/−11 (Caramia 2008) and to ~109 ka (Bini et al. 2020), respectively. At Poggio, two additional reference points of absolute chronology come from layer 9 and layer 3 of the shelter (TL 43,800 ± 3500 and ^14^C 11.630 ± 230, 14,026–13,108 cal. BP; Boscato et al. 2009, respectively) and concern the end of the Mousterian cycle and the end of the Epigravettian (Figure S1).

Available data on faunal association (Sala 1979) from layers 13–2 of the cave point to the predominance of red deer ( *Cervus elaphus* ) remains, followed by large bovids (*Bos/Bison*), roe deer ( *Capreolus capreolus* ), and caprine ( *Capra ibex* and *Rupicapra* sp.). Other taxa, such as proboscideans, equids, lagomorphs, and carnivores, are present. The fallow deer ( *Dama dama* ) is absent. Layer 18 of the shelter (Boscato et al. 2009) shares with the cave the absence of fallow deer, the dominance of red deer, and the abundance of bovids, as well as the occurrence of elephant and rhino. According to Boscato et al. (2009), these features, besides confirming the stratigraphic correlation of layer 18 with the deposit of the cave, adding biostratigraphic data to the cultural ones, allow an attribution to a cold climatic phase. Conversely, a change can be seen in layer 17 marked by the appearance of the fallow deer, which remains abundant throughout the Middle Paleolithic sequence, in keeping with milder climatic conditions recorded in the neighboring territories, and more generally in the southern Tyrrhenian area (e.g., Boscato et al. 2009; Sala 1983; Boschin et al. 2022; Spagnolo et al. 2024), which was recognized as a refugium not only for humans and animals but also for arboreal species (Di Pasquale et al. 2020; Boschin et al. 2022; see also Roditi et al. 2024).

The lithic assemblage from layers 13–3 is mainly composed of irregular flakes, generally short and wide, of small and very small size, obtained from local morphologically heterogeneous pebbles according to an expedient exploitation of cores (di Palma Cesnola 2001; Caramia 2008). The retouched tools are generally side scrapers and denticulates of poor technical quality. The occurrence of specimens from the Tayacoid tradition, such as Quinson‐type elements, hypercarinated side scrapers (planes or limaces), and Tayac points gives the industry of these layers an archaic appearance. A technological change is documented in layer 2 with the introduction of the Quina method both in the reduction system and in the type of retouching, which is often stepped (di Palma Cesnola 2001; Caramia 2008).

The molar, first called “find D” and later renamed to “Grotta del Poggio 1”, was collected in situ (Figure 2) in 1966 by one of the excavators directly from the deposit of layer 6, spit 1 (MIS 6; Palma di Cesnola's field notes, 7th May 1966; Figure S2). The history of the discovery of the human talus in 1964 is more problematic. Initially called “find E” and later renamed to “Grotta del Poggio 2” (Messeri and di Palma Cesnola 1976; Alciati et al. 2005), the talus was collected in the sediment at the bottom of the trench during the excavation of the Mousterian layer 4a and was therefore assigned to this stratigraphic unit (Palma di Cesnola's personal communication to AM). In describing the human finds, Messeri and di Palma Cesnola (1976) noted that the molar showed clear Neanderthal‐like features. On the other hand, the morphology of the talus was more puzzling, as it showed modern human features, which led Messeri and di Palma Cesnola (1976) to consider the possibility of the presence of morphologically modern individuals in the Mousterian period.

## 2 Materials and Methods

### 2.1 Data Acquisition

The molar tooth (Grotta del Poggio 1; hereafter GP1) was μCT scanned through high‐resolution μCT images at the Vienna Micro‐CT Lab, Department of Anthropology, University of Vienna (Vienna, Austria), with a Viscom X8060 μCT scanner using the following scan parameters: 120 kV, 100 μA, voxel size of 0.025 mm.

The talus (Grotta del Poggio 2; hereafter GP2) was μCT scanned at the Department of Physics and Astronomy, University of Bologna (Bologna, Italy), using an inhouse CT system (Kevex PXS10‐65 microfocus X‐ray tube and Varian PaxScan 2520D flat‐panel X‐ray detector; Albertin et al. 2019) at an isotropic voxel resolution of 0.040 mm (78 kV, 200 μA).

Three‐dimensional digital models of the talus and the tooth tissues (enamel and dentin) were obtained by segmenting μCT image data in Avizo 9.2 (Visualization Sciences Group Inc.). When necessary, 3D models were optimized and cleaned in Geomagic Desing X (3D Systems Software, Rock Hill, South Carolina, US).

As GP1 presents some cracks both on the enamel and the underlying dentin, a virtual restoration was conducted in Geomagic Design X in order to realign the tooth fragments and reduce the extent of the cracks on the crown that would compromise the subsequent analyses. We followed the guidelines from the protocols detailed in Vazzana et al. (2018) and Talamo, Fewlass, et al. (2021). For the crown, points of contact between the enamel fragments were identified and served as benchmarks for the alignment. After aligning the enamel fragments, these were joined. The same procedure was applied to the fragments of coronal dentin (Figure S3).

### 2.2 GP1

**FIGURE 3:**
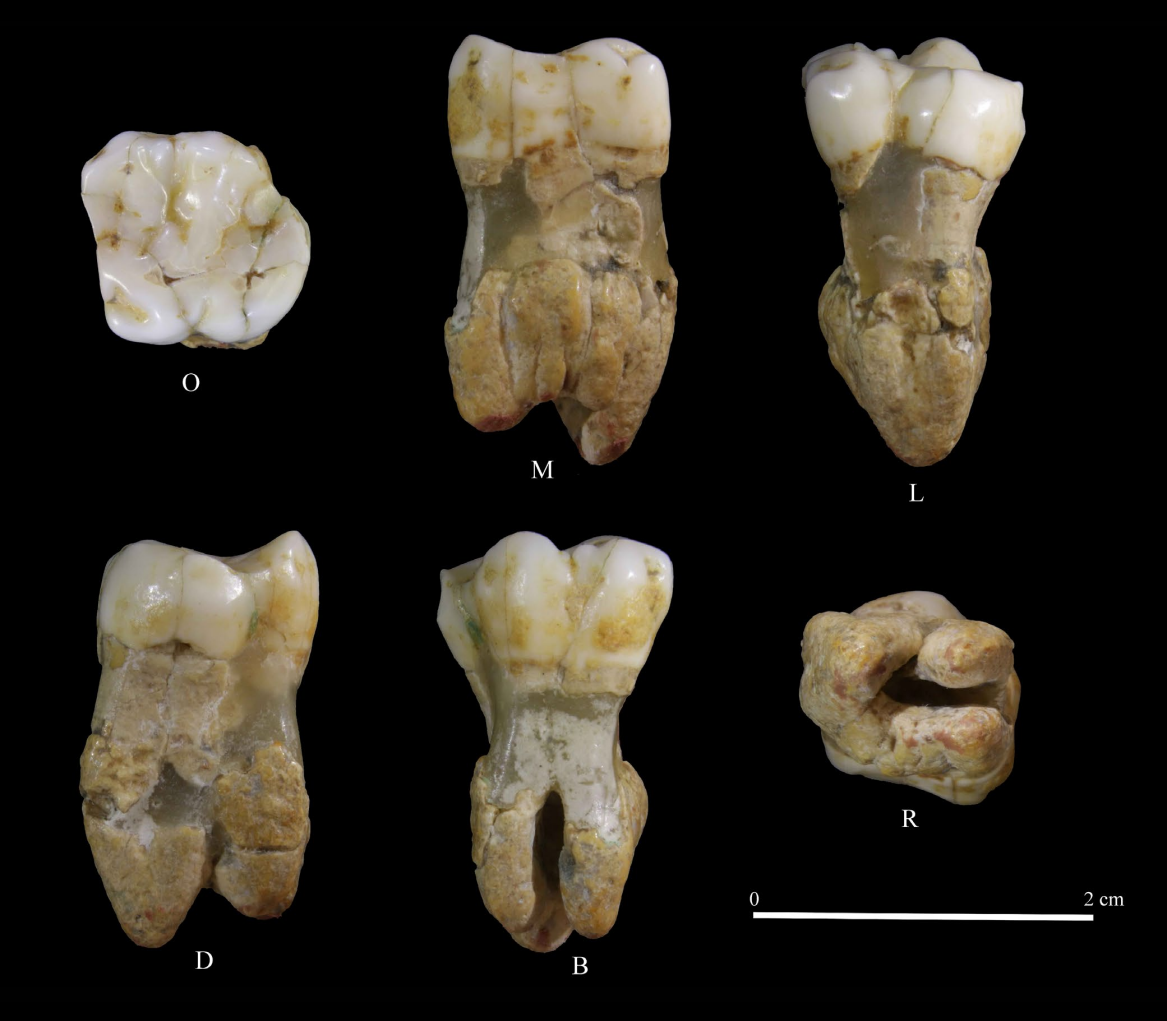
GP1. (O) Occlusal view; (M) mesial view; (L) lingual view; (D) distal view; (B) buccal view; (R) root view.

GP1 represents an upper left first molar (ULM1; Figure 3), whose morphometric description and evaluation were first conducted in the workflow of the methods adopted in this study.

On the 3D model of GP1, occlusal wear stage was assessed based on Molnar (1971). Non‐metric traits were observed both at the outer enamel surface (OES) and at the enamel‐dentin junction (EDJ), and recorded using the Arizona State University Dental Anthropology System (ASUDAS; Turner et al. 1991; Scott and Irish 2017).

In order to assess the metric traits of the tooth, we measured the mesio‐distal (MD) and bucco‐lingual (BL) crown diameters on its digital model by inscribing it in a bounding box. The BL and MD crown diameters were then compared with a sample of 93 individuals comprising recent *H. sapiens* (RHS; *n* = 26), Neanderthals (NEA; *n* = 30), Upper Paleolithic *H. sapiens* (UPHS; *n* = 25), and early *H. sapiens* (EHS; *n* = 12), obtained from Benazzi, Viola, et al. (2011) and references therein. A list of the samples used as comparison is provided in Table S1. The crown diameters of all specimens used in this analysis are provided in the Supporting Information.

A morphometric crown outline analysis based on geometric morphometrics (GM) methods was carried out on GP1 in order to shed light on its taxonomy, according to guidelines previously described for this tooth class (Bailey et al. 2014; Hublin et al. 2017). The comparative sample comprised 124 individuals, including recent *H. sapiens* (RHS; *n* = 80), Neanderthals (NEA; *n* = 18), Upper Paleolithic *H. sapiens* (UPHS; *n* = 19), and early *H. sapiens* (EHS; *n* = 7; Bailey et al. 2014). A list of the comparative samples is provided in Table S2.

First, the 3D digital model of the tooth was oriented in Geomagic Design X as follows: a spline curve was digitized on the cervical line of the tooth, and a best‐fit plane of the curve was computed. The tooth was aligned with the cervical plane parallel to the xy‐plane of the Cartesian coordinate system and rotated around the *z*‐axis so that the lingual side was parallel to the *x*‐axis. The crown outline was obtained by the projection onto the cervical plane (Benazzi, Douka, et al. 2011; Benazzi, Coquerelle, et al. 2011; Benazzi et al. 2012).

The outline of the oriented tooth was imported in Rhinoceros 5 Beta CAD environment (Robert McNeel and Associates, Seattle, WA) and centered superimposing the centroid of its area. Then, the outline was subdivided by 24 radial equiangularly distanced vectors having origin from the centroid. In particular, the subdivision started from the buccally directed and parallel to the y‐axis radius. The 24 pseudolandmarks were then scaled to the unit centroid size becoming Procrustes shape coordinates used to explore shape variation through principal component analysis (PCA; Benazzi, Douka, et al. 2011; Benazzi et al. 2012; Bailey et al. 2014, 2016, 2020; Harvati et al. 2019).

The GP1 pseudolandmarks lying on a worn‐out portion of the tooth crown due to the presence of the interproximal wear mesial facet (four pseudolandmarks) and on a cracked portion of the enamel (one pseudolandmark; Figure S4) were statistically estimated through the function “estimate missing” in the R package geomorph v4.0.0 (Adams et al. 2023) based on each mean of the four groups of the comparative sample (i.e., RHS, NEA, EHS, UPHS) and the overall sample (Figure S5). The five reconstructed crown outlines of GP1 were projected into the shape‐space of the comparative sample with the aim to evaluate if the reconstructions based on the reference mean could influence the final result. Since the choice of the reference group did not have any visible influence on the outcomes (Figure S6), we used the crown outline reconstructed on the overall mean of the comparative sample for the final analyses.

Although it is evident and extensively proved that a 2D GM crown outline analysis can be crucial in assessing the taxonomy of teeth with worn cusps (Benazzi, Coquerelle, et al. 2011; Benazzi et al. 2012; Bailey et al., 2014, 2020; Oxilia et al. 2022; Piccirilli et al. 2023), various examples in literature showed the combined use of GM analyses on the OES and the EDJ, suggesting that this approach could accomplish a reliable and comprehensive overview of the dental morphological variation (Morita et al. 2014; Hublin et al. 2017; Krenn et al. 2019; Šimková et al. 2021, 2024). With this in mind, we considered it advantageous to apply a 3D GM approach on the EDJ and the CEJ of GP1, too. We combined the EDJ and CEJ into one single 3D GM analysis, and we ran a single PCA on the (semi)landmarks representing both the curves. We followed Davies et al. (2024), by employing the (semi)landmark configuration proposed by them (Figure S7a), and used a comparative sample of Neanderthals (NEA; *n* = 13) and recent *H. sapiens* (RHS; *n* = 10) published therein. In addition, we included Krapina 136, Krapina 164 and Krapina 167, available from the Neanderthal Museum Digital Archive (former NESPOS; Quirin et al. 2024); Gibraltar 2, Qafzeh 10 and Qafzeh 15 from the ESRF Paleontology database (Smith et al. 2010); and 14 Iberian recent *H. sapiens* samples available from Morphosource (Gamarra et al. 2022). A list of the entire comparative sample is presented in Table S3.

Since the dentin horn tips of either GP1 and four comparative samples (Krapina 136, 164, and 167 and Qafzeh 10) were slightly flattened due to occlusal wear, a virtual reconstruction of the missing portions was conducted in Geomagic Design X, according to the procedure highlighted in Skinner (2008) and Davies et al. (2024). That was performed as an introductory step to obtain a more workable and realistic surface for the next phase, in which landmarks and semilandmarks were digitized on the 3D digital models of GP1 and the comparative samples in Viewbox v. 4 (dHAL software, Kifissia), by taking Davies et al. (2024) as reference. The coordinates from right teeth were mirrored on the x‐axis in order to have a comparative sample representing the left side.

The coordinates of the (semi)landmarks lying on the reconstructed portions of the new comparative samples were estimated with the function “estimate missing” in the R package geomorph v4.0.0 (Adams et al. 2023). KRP 136, KRP 164, and KRP 167 were estimated on the Neanderthal mean, while Qafzeh 10 was estimated on the *H. sapiens* mean.

The dentin horns reconstruction for GP1 was estimated with the same procedure described for the crown outline reconstruction. The estimation was based on each of the three groups of the comparative samples (i.e., RHS, EHS, and NEA) and on the overall sample. After that, the coordinates of (semi)landmarks were converted into Procrustes coordinates through the generalized Procrustes analysis (GPA; Gunz et al. 2005; Mitteroecker and Gunz 2009; Slice 2005) using the R package geomorph 4.0.0 (Adams et al. 2021). The Procrustes coordinates from the four reconstructions of the GP1 dentin were projected into the shape space of the comparative sample that showed no noticeable effect on the outcomes (Figure S8), leading us to use the reconstruction on the overall sample mean for the final analysis.

Then, a permutation test (R package Morpho v.2) on the first three principal component (PC) scores (*N* = 10.000, Holm adjustement method) was performed on the comparative sample to detect significant (*p* < 0.05) differences in crown outline, EDJ and CEJ shapes between groups. The number of PC scores used to perform the permutation test (i.e., the first 3 PC scores) was chosen based on the inflection point of the scree plot. Shapiro–Wilk tests were conducted on the first 3 PC scores (chosen with same selection criterion used for the permutation test) from the comparative groups to determine if they followed a normal distribution (Ghasemi and Zahediasl 2012), in order to meet the assumptions required for applying linear discriminant analysis (LDA). The normality assumption was violated for the crown outline data, then the “leave‐one‐out” cross‐validation quadratic discriminant analysis (QDA) was carried out. In contrast, the EDJ and CEJ data followed a normal distribution, so a LDA was run. Both QDA and LDA aimed to evaluate the probability of distinguishing Neanderthals from the *H. sapiens* groups, according to the crown outline and EDJ plus CEJ, respectively. For both QDA and LDA, we selected the minimum number of PCs that accounted for the highest accuracy, within the cut off of 70% to 90% of the total variation (Jolliffe 2002; Bailey et al. 2020; Sorrentino, Belcastro, et al. 2020). Tooth size was investigated as the centroid size (i.e., the square root of the summed squared distances between each landmark/semilandmark and the centroid of the landmark/semilandmark configuration) of both the EDJ and CEJ, visualized by means of a box plot.

Finally, we assessed whether the 2D and 3D morphological variation within Neanderthals was structured by geography or chronology, with the aim of identifying additional information relevant to interpreting our results. For geography, we tested correlations between the first 3 PC scores of the Neanderthal sample (including GP1) and specimen coordinates (latitude and longitude) using the Pearson product–moment correlation coefficient (r). We chose the first 3 PCs scores to perform this test based on the inflection point of the scree plot. Here, we examined the latitude and longitude separately to detect if one of them mainly drove any significant association (*p* < 0.05). Temporal structure was assessed by dividing specimens into two groups to overcome obstacles caused by the small sample size. We followed the rationale proposed by Profico et al. (2023), with adjustments based on our datasets: “early” Neanderthals (MIS 8–5 for 2D GM; MIS 6–5 for 3D GM), and “classic” Neanderthals (MIS 4–3). For the first 3 PC scores (selected as the third PC score was related to the inflection point of the scree plot), we tested the assumptions of normality (Shapiro–Wilk test). When this assumption was met, one‐way ANOVA was applied to compare PC scores across MIS groups and detect any significant (*p* < 0.05) differences; otherwise, the non‐parametric Kruskal‐Wallis test was used. In case of significant results, the relative positions of Neanderthal with respect to their geography and chronology were visualized within the original PCA plots. Geographical and chronological data of Neanderthals are in Tables S4 and S5.

The statistical analyses were performed using R v.4.2.2 (R Development Core Team 2021).

### 2.3 GP2

**FIGURE 4:**
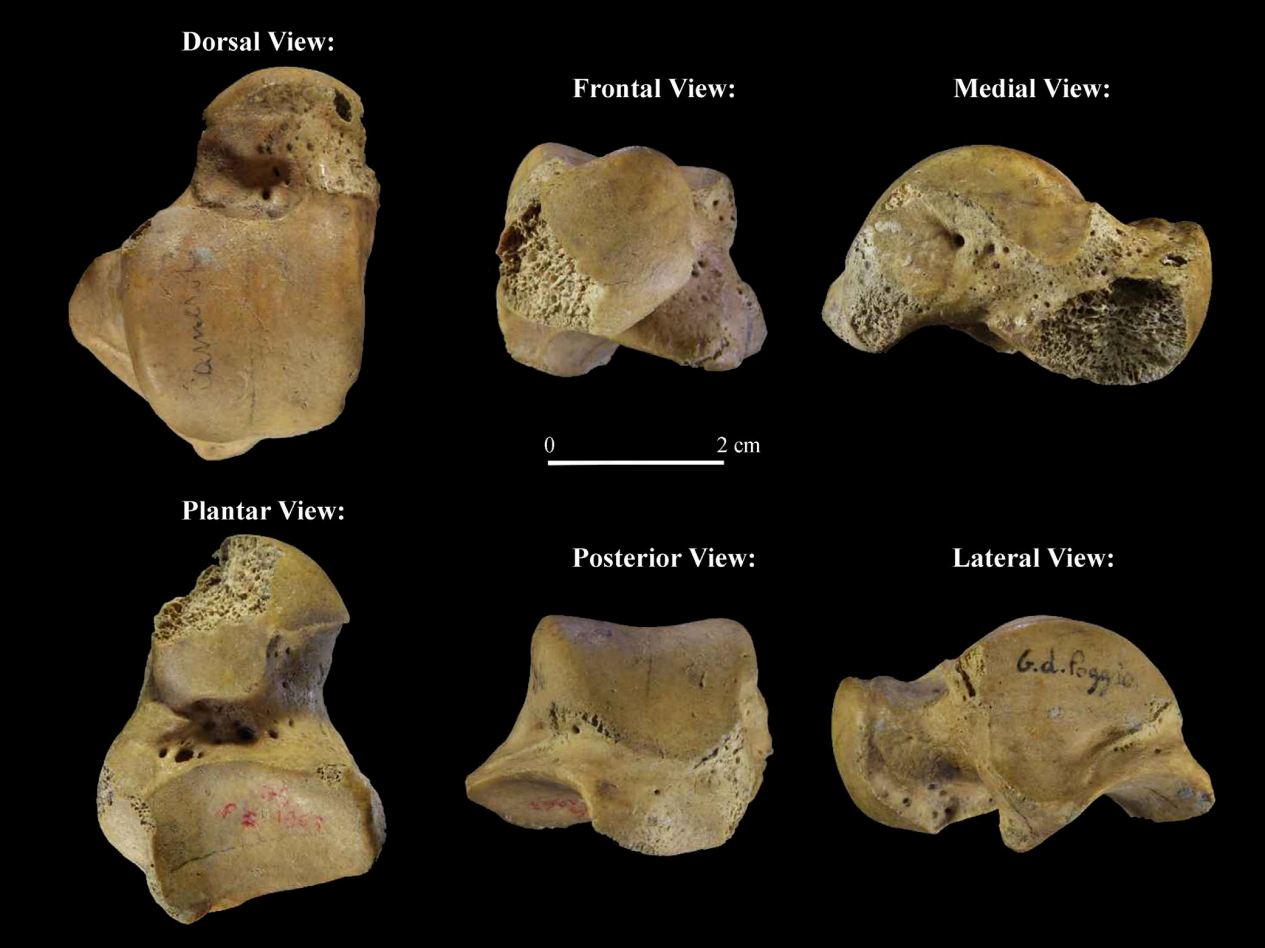
GP2.

GP2 represents a left talus of an adult individual based on the developmental patterns observed in modern humans, on the complete development of the bone and the articular facets, and on the general aspect of the bone (Figure 4). The talus is well preserved except for a missing portion of the right side of the talar neck and head and a slight erosion affecting also the medial posterior tubercle.

The shape of GP2 was compared with a published comparative sample of left tali from Neanderthals and *H. sapiens* (Sorrentino, Stephens, et al. 2020; Sorrentino et al. 2021, 2022). The Neanderthal sample includes Krapina 235 and Krapina 237, La Chapelle‐aux‐Saints, La Ferrassie 1 and La Ferrassie 2, Tabun C1, Spy 2, Shanidar 5, Amud 1, and Regourdou 1 (Sorrentino et al. 2021). The *H. sapiens* sample includes 39 individuals from Bologna (Italy) of the post‐industrial revolution period (Belcastro et al. 2017; Sorrentino, Belcastro, et al. 2020), 15 Holocene hunter‐gatherer individuals from the Black Earth site (Carrier Mills Archaeological District, Illinois, USA) of the Late Archaic period (~4950 cal BP; Jefferies 2013), and 7 Upper Paleolithic hunter‐gatherer individuals from Italy (Veneri 2, Romito 7, Romito 8, Romito 9, Paglicci 164, Paglicci 25, and Villabruna) of the Gravettian and Epigravettian periods (Alciati et al. 2005).

The talar shape was analyzed using a 3D template of 251 landmarks and semilandmarks described in (Figure S7b; Sorrentino, Belcastro, et al. 2020; Sorrentino, Stephens, et al. 2020; Sorrentino, Carlson, et al. 2020), using Viewbox v. 4 (dHAL software, Kifissia). The missing portion of the right side of the Poggio talar neck and head was estimated based on the Neanderthal mean and the *H. sapiens* mean, respectively, using the function “estimate missing” in the R package geomorph v. 4.0.0 (Adams et al. 2023). Translated, scaled, and rotated coordinates (i.e., Procrustes coordinates) were computed by the generalized Procrustes analysis (GPA). At the same time, semilandmarks were allowed to slide with each recursive update of the Procrustes mean (Gunz et al. 2005; Mitteroecker and Gunz 2009; Slice 2005) using the R package geomorph 4.0.0 (Adams et al. 2021). For the comparative sample, shape coordinates were used to perform the PCA. The PC scores of GP2 reconstructed on the Neanderthal and *H. sapiens* means, respectively, were predicted by multiplying their matrix of shape variables with the covariance matrix of the known sample, using R‐code provided in Benazzi, Bookstein, et al. (2011), Lugli et al. (2022), Sorrentino, Belcastro, et al. (2020); Sorrentino, Carlson, et al. (2020); Sorrentino et al. (2022, 2023). Then, the predicted PC scores were projected into this shape space to assess its morphological variation in comparison to *H. sapiens* and Neanderthal groups, while validating the influence of the two reconstructions. Centroid size was used as a proxy of the talar size, and a box plot was used to visualize the variation of the GP2 in respect to the comparative sample.

Furthermore, a between‐group PCA was conducted in order to reduce the number of PCs (obtaining 3 PCs in case of four groups of the comparative sample) using the function “groupPCA” in the R package Morpho v.2.8 (Schlager 2017), predicting the group PC scores of GP2 reconstructed on the Neanderthal and *H. sapiens* means, respectively. This allows us to overcome issues related to sample size, that is, ensuring that the smallest group has more cases than the number of PCs to be used in LDA or QDA to achieve 70% to 90% of total variance (Jolliffe 2002). Therefore, LDA was performed on the three group PCs as all PCs per group are normally distributed (Shapiro test), to evaluate the posterior probability (P_post_) of taxonomic assignment of Poggio reconstructed tali to Neanderthal or *H. sapiens* (Upper Paleolithic hunter‐gatherers, Holocene hunter‐gatherers, Post‐industrial individuals). The statistical analyses were conducted in R v.4.2.2 (R Development Core Team 2021).

### 2.4 Absolute Dating

Absolute dating was not performed on the molar, as any invasive methods were allowed by the Archaeological Office due to the importance and unicity of this remain. Thus, absolute dating was performed only on the talus and rib. Collagen was extracted from the talus and rib samples from Grotta del Poggio at the Department of Human Evolution, MPI‐EVA, following the protocol outlined in Talamo and colleagues (2021). The sample was demineralized in 0.5 M HCl at 4°C until soft and the CO_2_ effervescence had ceased, with HCl being replaced twice per week. After demineralization, the sample was treated with 0.1 M NaOH for 30 min to remove humic acid contamination, followed by re‐acidification in 0.5 M HCl. The sample was then gelatinized in pH 3 HCl at 75°C for 20 h, filtered to remove particles larger than 60–90 μm (Ezee filters, Elkay Labs, UK), and ultrafiltered to isolate the > 30 kDa fraction (Sartorius VivaSpin Turbo 15). Filters were pre‐cleaned prior to use (Talamo, Nowaczewska, and Picin 2021). The > 30 kDa fraction was lyophilized for 48 h, and the resulting collagen was weighed to determine its yield as a percentage of the dry sample weight.

The suitability of the extract for dating was evaluated based on collagen yield (minimum ~1%) and elemental values (Klinken van 1999; Talamo, Nowaczewska, and Picin 2021). The extract exhibited characteristics of well‐preserved collagen (Table 1) and was thus submitted for accelerator mass spectrometry (AMS) dating. The collagen extract was sent to the Curt‐Engelhorn‐Center for Archaeometry in Mannheim, Germany (CEZA, lab code: MAMS), where it was combusted to CO_2_ in an elemental analyzer (EA), converted catalytically to graphite, and subsequently measured on a MICADAS AMS (Kromer et al. 2013).

**TABLE 1:** Radiocarbon dating results for a rib fragment and a talus from Grotta del Poggio.

Sample name	Description	MPI code	Sample taken (mg)	Collagen (mg)	% of collagen	δ^13^C	δ^15^N	%C	%N	C:N	AMS code	^14^C Age	1*σ* Err	Cal BC/ad 68.2%	Cal BC/ad 95.4%
Poggio RIM ‘74	Rib fragment	R‐EVA 3332	590	33.4	5.66	−19.6	8.3	41.7	15.6	3.1	MAMS‐43282	3891	21	2456–2345	2462–2297
Poggio E astragalo	Sample of talus	R‐EVA 3333	423.3	33.6	7.94	−20.1	7.4	43.2	15.4	3.3	MAMS‐43283	3273	20	1599–1504	1613–1499

*Note:* The table includes sample descriptions, laboratory codes, collagen yield and quality indicators (δ^13^C, δ^15^N, %C, %N, C:N), radiocarbon ages with 1*σ* errors, and calibrated date ranges at 68.2% and 95.4% probability. Calibration was performed using the IntCal20 calibration curve and the OxCal 4.4 program (Ramsey 2009; Reimer et al. 2020).

## 3 Results

### 3.1 GP1

#### 3.1.1 Morphological Description

GP1 is a taurodont upper left first molar (ULM1) showing well‐developed and very robust roots with three free apexes. The roots are almost totally fused on the mesial and distal face; on the buccal face, the fusion covers half of the roots. They were originally highly damaged and underwent a restoration that allowed to reconstruct their morphology (Figure 3). Additionally, some severe fractures of the enamel affect protocone and paracone and are well visible in the occlusal view; thus, a virtual restoration was conducted (Figure S3). As a result, the restored tooth crown has a MD diameter of 13.60 mm and a BL diameter of 12.91 mm, while the original diameters before the virtual restoration were 13.81 mm (MD) and 12.93 mm (BL).

The occlusal enamel surface is slightly worn out, with a weak exposure of dentin, equal to wear stage 3 following Molnar (1971). The mesial interproximal wear facet is broad (length = 7.31 mm; height = 4.33 mm), while the distal interproximal facet is smaller (length = 3.3 mm; height = 2.97 mm), albeit both of them are altered by the presence of enamel cracks. The four principal cusps (protocone, paracone, metacone, and hypocone) are strongly expressed; in particular, the metacone corresponds to ASUDAS grade 3.5 and the hypocone to ASUDAS grade 5. The hypocone wall crown bulges as it shows a bucco‐distally pronounced crown convexity, with a subsequent rhomboidal shape of the occlusal surface. In addition to the four principal cusps, the tooth also shows metaconule, represented by a small cuspule (ASUDAS grade 3); a parastyle (ASUDAS grade 1); moderate mesial marginal tubercles (MAT) on the dentin (grade 1 in Scott and Turner 1997); and an accentuated accessory ridge (AC‐PAR) on the paracone. The Carabelli's trait is represented by a moderate cusp with a free apex (ASUDAS grade 6; Figure S9). Small traces of calculus are present on the lateral sides of the crown (Figure 3).

The scatterplot of BL and MD crown diameters is shown in Figure S10. *H. sapiens* specimens overlap with most of the distribution of the Neanderthal specimens. Nevertheless, towards the right side of the plot, when MD diameter increases, most of the specimens tend to belong to Neanderthals. GP1 falls within this area, although in a marginal location due to its large MD diameter. According to this analysis, GP1 is more similar to the Neanderthal range.

#### 3.1.2 GM Analysis

**FIGURE 5:**
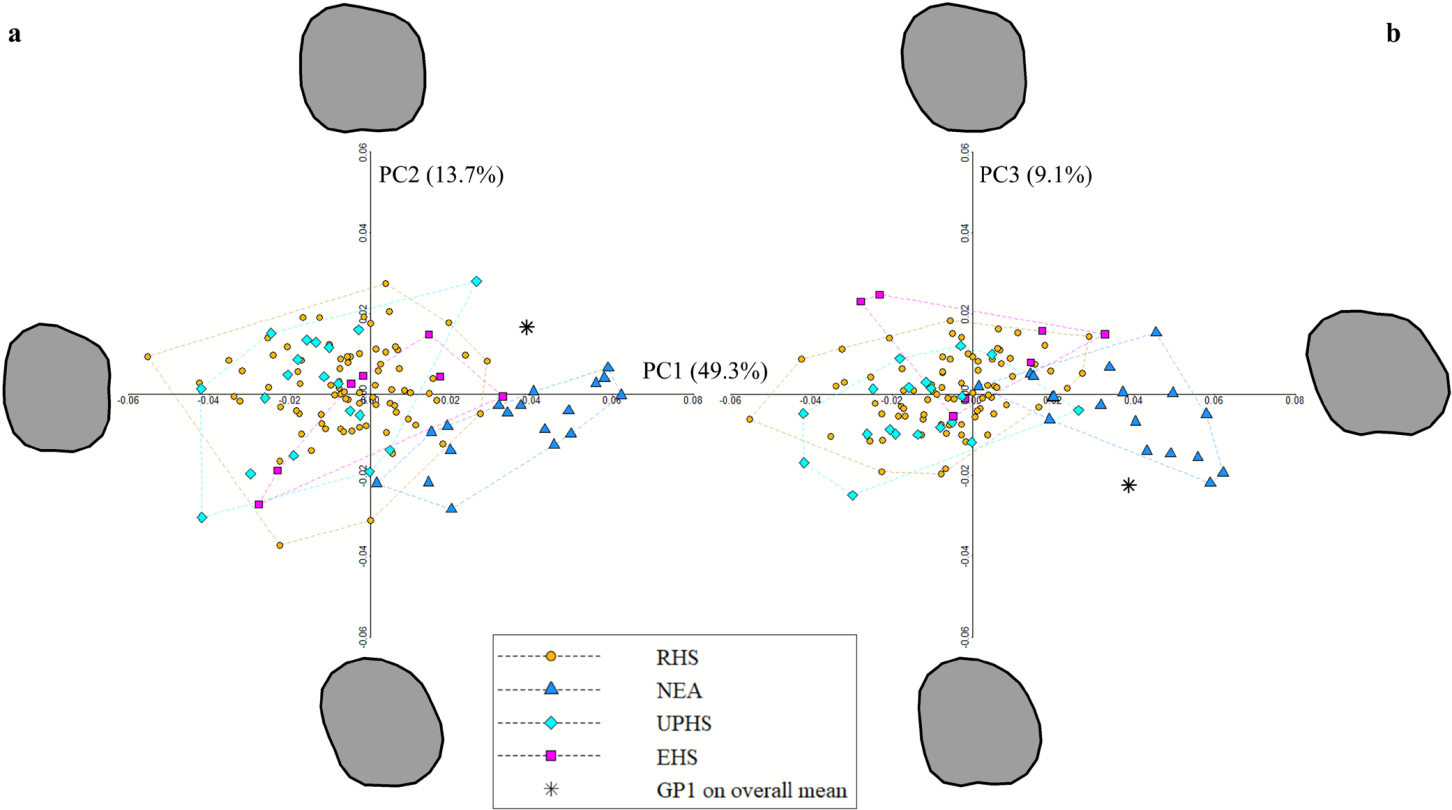
Principal component analysis (PCA) plot of GP1 crown outline. (a) PC1 vs. PC2; (b) PC1 vs. PC3. The magenta asterisk represents GP1 reconstructed on the overall mean projected in the morpho‐space made by recent *H. sapiens* (RHS); Neanderthals (NEA); Upper Paleolithic *H. sapiens* (UPHS); early *H. sapiens* (EHS).

The crown outlines shape‐space is shown in Figure 5. The first 3 PCs account for 72.1% of the total variance, of which 49.3% is represented by PC1, 13.7% by PC2, and 9.1% by PC3. Neanderthals separate from the *H. sapiens* specimens (i.e., RHS, UPHS, and EHS) plotting mainly along PC1 positive due to the increasingly rhomboid shape characterized by a protruding and bulging hypocone. Furthermore, Neanderthals plot mainly along the negative values of PC2, where crown outline shape becomes less symmetrical. There is not a clear separation among RHS, UPHS, and EHS along both PC1 and PC2, whereas a separation between EHS and UPHS can be better detected along PC3. In PC3, both *H. sapiens* and Neanderthals are distributed on the positive and negative values of PC3. GP1 falls within the positive PC1 and PC2 values, between Neanderthals and *H. sapiens* (Figure 5a). Particularly, when observing the PC1 and PC3 values, GP1 is closer to the Neanderthal range than to the *H. sapiens* range (Figure 5b).

The permutation test (*N* = 10.000, Holm adjustment method; Table S6) conducted on the first 3 PC scores (72.1% of the total variance) showed a statistically significant difference between NEA and the *H. sapiens* groups (NEA‐EHS, *p* = 0.001; NEA‐UPHS, *p* = 0.0006; NEA‐RHS, *p* = 0.0006).

After the Shapiro–Wilk test (PC1: *p* = 0.405; PC2: *p* = 0.449; PC3: *p* = 0.712 for NEA; PC1: *p* = 0.781; PC2: *p* = 0.027; PC3: *p* = 0.886 for pooled *H. sapiens* ), the QDA performed on the first three PCs discriminated Neanderthals from *H. sapiens* with an accuracy of 95.2% (Table S7). GP1 is classified as Neanderthal with a posterior probability of 94.4%. The Pearson's product–moment correlation on the first three PC scores (Table S8) revealed no significant association with longitude or latitude within the Neanderthal sample. After confirming normality of data distribution for the first three PCs, one‐way ANOVA showed no significant differences in any of the first three PC scores of Neanderthals grouped by MIS stage (PC1: *p* = 0.089; PC2: *p* = 0.254; PC3: *p* = 0.24).

**FIGURE 6:**
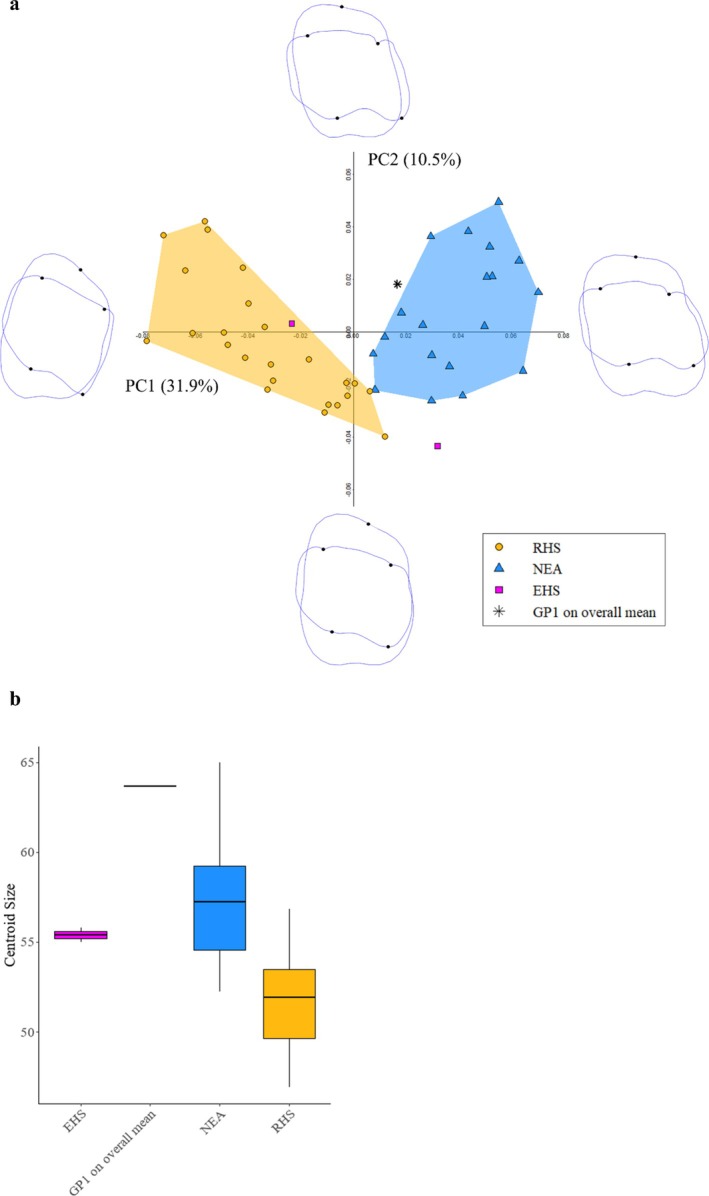
Results of the 3D GM analysis of GP1 EDJ and CEJ. (a) PCA 2D plot showing PC1 versus PC2. The black symbols represent GP1 reconstructed on the overall mean projected in the morpho‐space made by recent *H. sapiens* (RHS); Neanderthals (NEA); Upper Paleolithic *H. sapiens* (UPHS); early *H. sapiens* (EHS). Extreme shapes along each principal component represent the mean shape of the sample warped along the respective PC axis by the maximum observed PC score in each direction. (b) Boxplot of the centroid size for GP1 and the comparative sample. The median is represented by the horizontal line inside the box. The interquartile range is the colored box, covering the middle 50% of values. The vertical whiskers extend to the minimum and maximum values that are not outliers.

The shape‐space PCA of the EDJ and CEJ are shown in Figure 6. The first three PCs reach the 51.8% of the total variance (PC1 = 31.9%, PC2 = 10.5%, and PC3 = 9.4%). Neanderthals and *H. sapiens* are separated along PC1, with Neanderthals being located exclusively along the positive values of PC1, while *H. sapiens* placed mainly along its negative values. GP1 is situated within the positive PC1 values, in the immediate proximity of the Neanderthal range (Figure 6a). The permutation test (*N* = 10.000, Holm adjustment method; Table S9) performed on the first three PC scores (51.8% of the total variance) showed a statistically significant difference between NEA and RHS (*p* = 0.0003). The Shapiro–Wilk tests showed that both the pooled *H. sapiens* (PC1: *p* = 0.915; PC2: *p* = 0.146; PC3: *p* = 0.906) and NEA (PC1: *p* = 0.494; PC2: *p* = 0.486; PC3: *p* = 0.555) groups are normally distributed, therefore a LDA was conducted. The results of the LDA (Table S10) performed on the first seven PCs (74.9% of variance explained) discriminate *H. sapiens* from Neanderthals with an accuracy of 100%, and GP1 is classified as Neanderthal with a posterior probability of 100%. The Pearson's product–moment correlation on the first three PCs (Table S11) showed significant association between PC1 (*p* < 0.001) and PC2 (*p* = 0.032) with the longitude within the Neanderthal sample. No correlation was found with the latitude. In the PC1 versus PC2 plot (Figure S11), Croatian samples are located along the most positive values of PC1 and PC2, whereas GP1 and the westernmost individuals fall in the area of the lowest values of PC1. After demonstrating normality of data distribution for the first three PCs, one‐way ANOVA revealed significant differences in PC1 (*p* = 0.007) and PC2 scores (*p* = 0.031) of Neanderthals grouped by MIS stage, while no significance for PC3 (*p* = 0.253). The “classic” Neanderthals (MIS 4–3) are more closely clustered within the lowest values of PC1, and they mostly fall within the negative PC2 values. Differently, the “early” Neanderthals –GP1 included– are scattered on a wider area of the positive PC1 values, spreading mostly along the positive scores of PC2 values (Figure S12).

When considering its centroid size, GP1 is a large‐size tooth, exceeding the higher quartile of Neanderthals, but staying within their range (Figure 6b).

### 3.2 GP2

#### 3.2.1 GM Analysis

**FIGURE 7:**
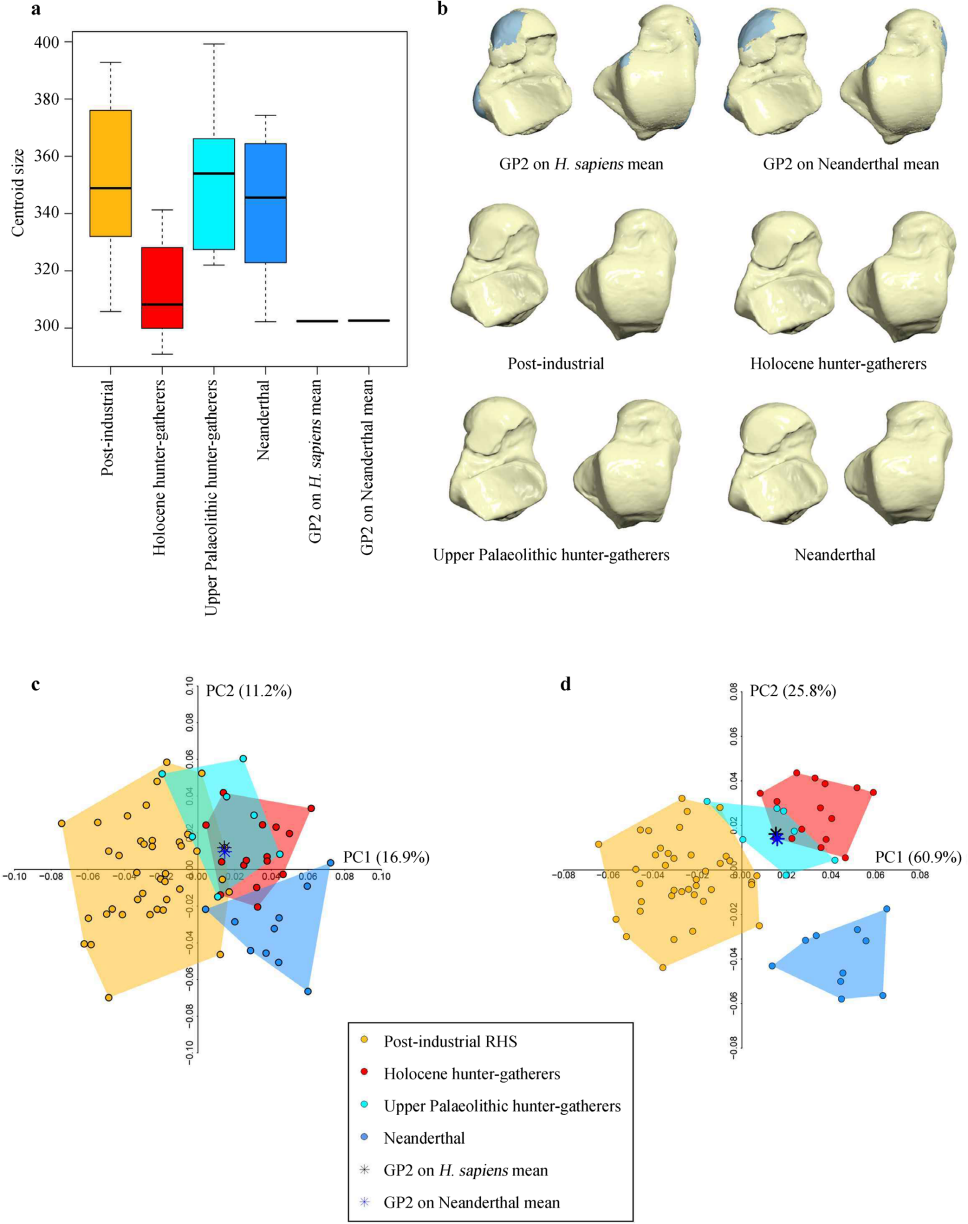
Results of the 3D GM analysis on GP2. (a) Boxplot of the centroid size for GP2 and the comparative sample; (b) GP2 reconstruction on *H. sapiens* and Neanderthal mean (first row), and mean shapes for the comparative samples (second and third row); (c) principal component analysis (PCA) plot of GP2 whole shape; (d) group‐PCA plot of GP2 whole shape. The black asterisk represents GP2 reconstructed on *H. sapiens* mean, and the blue asterisk represents GP2 reconstructed on Neanderthal mean, both projected in the morpho‐space made by Post‐Industrial recent *H. sapiens* (RHS; yellow), Holocene hunter‐gatherers RHS (red), Upper Paleolithic hunter‐gatherers HS (cyan); Neanderthals (light blue).

GP2 is a relatively small talus when compared with the comparative sample, falling within the lower quartile of Holocene hunter‐gatherers (Figure 7a). GP2 reconstruction on Neanderthal and *H. sapiens* mean, respectively (Figure 7b), does not affect the final results in both PCA (Figure 7c) and between‐group PCA (Figure 7d) as both the reconstructions plot very close to each other. The shape space PCA and group‐PCA plots show that talar shape separates Neanderthals from *H. sapiens* groups, as previously observed by Sorrentino et al. (2021). Among *H. sapiens* groups, a clear separation is observed along PC1 between post‐industrial individuals of Bologna and both Holocene and Upper Paleolithic hunter‐gatherers, and this separation is emphasized in the group‐PCA (Figure 7d). The two reconstructions of GP2 plot in the area of overlap between Holocene and Upper Paleolithic hunter‐gatherers. They resemble these groups in several morphological traits: the increased dorsal convexity of the trochlea, a mediolaterally wider anterior margin of the trochlea with an anterior extension of the medial margin, a lateral displacement of the lateral malleolar facet, and a more concave posterior calcaneal facet (Figure 7b).

Cross‐validation LDA was highly accurate using the first three group‐PCs (98.6%) with all groups being classified with 100% accuracy except for post‐industrial individuals classified with 97.4% accuracy. GP2 reconstructed on either the *H. sapiens* or Neanderthal mean is classified as Holocene hunter‐gatherer, with P_post_ values of 98.4% and 97.6%, respectively.

#### 3.2.2 Radiocarbon Dating

The radiocarbon dating of the talus yielded a calibrated age range of 1599–1504 bc at 68.2% probability, and 1613–1499 bc at 95.4% probability, thus locating this individual in the Middle Bronze Age (Table 1).

For comparison with the talus, a radiocarbon dating on the human rib fragment from the metal age deposit excavated in 1974 was performed and yielded a calibrated age range of 2456–2345 bc at 68.2% probability, and 2462–2297 bc at 95.4% probability (Table 1).

## 4 Discussion and Conclusion

In the present study, we provide the taxonomic reassessment of the ULM1 (GP1) and the left talus (GP2) from Grotta del Poggio. As highlighted in Palma di Cesnola's field notes, the tooth was found in situ in layer 6, while the talus was found in the collapsed sediment during the excavation of layer 4a and therefore hypothesized to derive from this layer.

The 3D GM analysis attributed the talus to *H. sapiens* . Particularly, both PCA and between‐group PCA show that the GP2 reconstructed on the *H. sapiens* mean and on the Neanderthal mean plot within Upper Paleolithic and Holocene hunter‐gatherers and assigned to them with a P_post_ of 97.6%. This confirms the morphological examination of Messeri and di Palma Cesnola (1976) who had noticed the modern morphology and metrics of the talus. The ambiguity surrounding the chronological attribution of this specimen, due to its retrieval not in situ, was clarified through radiocarbon dating performed directly on a bone sample from GP2. With a calibrated age range of 1599–1504 cal BC at 68.2% probability and 1613–1499 cal BC at 95.4% probability, GP2 is confirmed to belong to a recent *H. sapiens* , specifically from the Middle Bronze Age. The systematic use of the upper part of the cave as a burial place during the Metal Ages is further confirmed by the radiocarbon chronology of the human rib fragment which yielded calibrated values comprised between 2456–2345 (68.2%) and 2462–2297 (95.4%) BC, allowing to ascribe this individual to the end of the Copper Age/Early Bronze Age.

As for the tooth, our newly generated digital data unanimously point to an attribution to *H. neanderthalensis*, in accordance with the first macroscopic observation of Messeri and di Palma Cesnola (1976). The crown morphology suggests an attribution to Neanderthals, due to the presence of some typical Neanderthal features, such as taurodontism and the highly expressed and protruding hypocone causing the rhomboidal shape of the occlusal surface (Bailey 2004; Gómez‐Robles et al. 2007; Kupczik and Hublin 2010; Benazzi, Viola, et al. 2011; Benazzi, Nguyen, et al. 2015). The BL and MD crown diameters place GP1 close to a Neanderthal individual, although these diameters do not provide a clear taxonomic separation between Neanderthals and *H. sapiens* , as already shown in the existing literature (Bailey and Hublin 2005; Benazzi, Viola, et al. 2011). Conversely, the 2D GM analysis of the crown outline is known to offer a reliable outcome of the morphological variability between *H. sapiens* and Neanderthals (Benazzi et al. 2012; Bailey et al. 2014, 2020). Based on the PCA plot, GP1 mainly resembles the Neanderthal crown outline, especially in the rhomboid shape and the pronounced hypocone. This is confirmed by the results of the QDA that classify the GP1 as Neanderthal with a P_post_ of 94.4% (Table S5). The 3D GM analysis of the EDJ and CEJ unambiguously attribute GP1 to Neanderthals, with a posterior probability of 100% (Table S8). In addition, the size of GP1 is within the Neanderthal range, close to its upper bound. The ascription of GP1 to Neanderthals is clear from the P_post_ obtained by the QDA of both the 2D and 3D GM analyses. That allows further consideration of its relative position with respect to the other Neanderthal specimens in the PCA plots, as this result may potentially reflect the presence of a chronological or geographical pattern in the morphological variation of Neanderthal teeth. Regarding the crown outline variability, no significance was found, while stronger evidence comes from the 3D GM data. The morphological variation within Neanderthal specimens is structured by geography, with the longitude significantly influencing the outcome. The Neanderthals exhibit a spatial pattern consistent with a west–east gradient. That, however, should be interpreted with caution, as our comparative sample is affected by an intrinsic bias: whereas the western individuals come from multiple sites and countries, those from further east (i.e., the Croatians specimens) all derive from the same Krapina population, which likely accounts for their similarity. Future studies could compare these results with Neanderthal specimens from eastern Europe and western Asia, thereby increasing the variability among eastern Neanderthals.

The attribution of GP1 to Neanderthals is in line with its finding in the Middle Paleolithic deposit of layer 6. The geological and biostratigraphic‐based time allocation of layer 6 is corroborated by the TL dating of layer 17 in the adjacent shelter, which sets the *terminus ante quem* for the cave's series at 111.800 ± 9.500 BP, also confirming that GP1 was older than this date (Gambassini and Ronchitelli 1998; Boscato et al. 2009). Notably, we detected a chronological structure in the 3D morphological variation of Neanderthals. The “early” Neanderthals (MIS 6–5) cover a much wider range of variability than the “classic” Neanderthals (MIS 4–3). That could suggest that “early” Neanderthals retained greater morphological diversity, which later decreased in the “classic” Neanderthals, resulting in a more stable pattern. This interpretation is consistent with previous evidence from other anatomical districts of the Neanderthal skeleton (Urciuoli et al. 2025). Considering the position of GP1, we hypothesize that its slight deviation from the range of the comparative Neanderthals in the GM analyses including *H. sapiens* likely reflects normal intra‐specific variation and contributes updating the ongoing debate about the extent of Neanderthal dental morphological variability (Benazzi, Viola, et al. 2011; Benazzi et al. 2013).

Based on current knowledge, there is only one other specimen as ancient as the Poggio tooth in southern Italy, namely the Altamura fossil, dated to 130.1 ± 1.9–172 ± 15 ka (Lari et al. 2015; Di Vincenzo et al. 2019; Profico et al. 2023; Buzi et al. 2024). In the south of the peninsula, the frequency of Neanderthal finds increases slightly from the beginning of MIS 5 and especially after the last interglacial (MIS 5e; Issel 1914; Hearty et al. 1986; Asioli et al. 2005; Pasquetti et al. 2021), although the stratigraphic contexts and chronology are not known for all of them. If we only consider human remains with reliable chronologies, we can distinguish a first group chronologically referable to MIS 5d‐a. This is composed of the mandible from Riparo del Molare di Scario (layer 51; Campania; Mallegni and Ronchitelli 1989), possibly the four teeth from Grotta Taddeo in the immediate vicinity of Poggio, including an upper right first molar with similar morphological features to GP1, such as the rhomboidal occlusal outline and the highly expressed Carabelli's trait (Campania; Benazzi, Viola, et al. 2011); in Calabria, a parietal bone from Nicotera (Calabria; Bonfiglio et al. 1986) and three teeth from Grotta del Cavallo (layers M2f, M2a and L; Apulia; di Palma Cesnola and Messeri 1967; Fabbri and Vincenti 2020—for a different view on the chronological position of these layers, see Sarti and Martini 2020). A second group is dated to the end of the Mousterian cycle (MIS 4–3) and consists of two teeth from Roccia San Sebastiano (Campania; Oxilia et al. 2022) and Grotta del Cavallo layer FIIIb (Apulia; Fabbri et al. 2016), and a mandible from Archi layer C‐3 (Calabria; Mallegni and Trinkaus 1997).

Considering the above mentioned sites and their presence in the broader perspective of the Italian Middle Paleolithic (Alciati et al. 2005; Buzi et al. 2021) from both a chronological and quantitative point of view, it is clear that more emphasis should be placed on the fact that GP1 is among the earliest rare Neanderthal specimens for the Italian territory (the oldest being the skulls from Saccopastore in Lazio, with an age of ~250 ka—Marra et al. 2015) and, together with the Altamura fossil, the earliest for southern Italy.

Unfortunately, a morphological comparison between GP1 and the UM1 of the Altamura specimen is severely hampered by the inaccessible position of the skeleton inside the Grotta di Lamalunga, causing a scarce availability of information about its dental features (Riga et al. 2020). According to Riga et al. (2020), the impossibility of observing any trace of taurodontism in the Altamura maxillary molars may not reflect actual absence, but rather the presence of a concretion layer covering the roots and thus precluding the examination. Since taurodontism was identified in the lower molars, its presence in the upper molars cannot be ruled out, potentially representing an additional similarity with GP1.

In conclusion, the digital morphometric analyses of GP1 and GP2, together with the direct radiocarbon dating of GP2, contribute to finally clarify their taxonomic assessment. Our results support the attribution of GP1 to Neanderthal and show that GP2 belongs to *H. sapiens* , in agreement with earlier observations. Our direct radiocarbon dating clearly establishes GP2 as a modern human from the Middle Bronze Age, confirming its provenance from a more recent layer than the Mousterian layer 4a. Therefore, its presence in the existing literature on Paleolithic fossil hominins should be revised (Alciati et al. 2005; Buzi et al. 2021) and it should be removed from any comparative samples of fossil hominins for morphological and morphometric analyses of the talus.

## Author Contributions

Erica Piccirilli: conceptualization, formal analysis, data curation, investigation, methodology, validation, writing – original draft, writing – review and editing, visualization, software. Rita Sorrentino: conceptualization, formal analysis, data curation, investigation, methodology, validation, writing – original draft, writing – review and editing, visualization, software. Francesca Seghi: visualization, writing – review and editing. Antonino Vazzana: methodology, software, writing – review and editing. Maria Giovanna Belcastro: resources, writing – review and editing. Sahra Talamo: methodology, validation, formal analysis, investigation, resources, data curation, writing – original draft, writing – review and editing, visualization, software. Katerina Harvati: writing – review and editing. Matteo Bettuzzi: data curation, writing – review and editing. Maria Pia Morigi: resources, data curation, writing – review and editing. Gerhard Weber: resources, writing – review and editing. Giulia Capecchi: writing – review and editing. Vincenzo Spagnolo: visualization, writing – review and editing. Ivan Martini: writing – original draft, writing – review and editing. Adriana Moroni: conceptualization, data curation, funding acquisition, project administration, resources, supervision, writing – original draft, writing – review and editing, visualization. Francesco Boschin: writing – original draft, writing – review and editing. Stefano Ricci: writing – original draft, writing – review and editing. Stefano Benazzi: conceptualization, data curation, investigation, resources, supervision, validation, funding acquisition, project administration, writing – original draft, writing – review and editing.

## Funding

K.H. is also supported by the Carl Friedrich von Siemens Foundation. Past research and fieldwork at Grotta del Poggio were funded by the University of Siena. Current research and excavations have been funded by the National Geographic Society/Exploration Grant Program (grant NGS‐61617R‐19; PI: I. Martini), the ERC AdG n. 101019659—FIRSTSTEPS (PI: K.H.) and the European Union–Next Generation EU PRIN 2022 TRACE project (awarded to Stefano Benazzi and Adriana Moroni).

## Conflicts of Interest

The authors declare no conflicts of interest.

## Supporting information


**Figure S1:** Poggio. Stratigraphic correlations between Cave, shelter and niche. On the left: stratigraphic detail of layers 18 to 12 of the shelter in which the top surface of layer 18 can be seen sealed by a stalagmitic crust tentatively referred to MIS5e on the basis of a TL date (111.800 ± 9.500) obtained in the following layer 17 (from Gambassini's archive modified). The series of Poggio lies on a marine conglomerate 9.5 m asl attributed to MIS7, on geological and biostratigraphic grounds. Layers 18 of the shelter and layers 13–2 of the cave contain pre‐Levallois industries, whilst the lithic assemblages of layers 17 to 9 of the shelter are characterized by the employment of the Levallois method. Layers 7 to 2 of the shelter have yielded Epigravettian industries (Boscato et al. 2009).

**Figure S2:** Excerpt from Palma di Cesnola's excavation field notes, dated May 7th, 1966, documenting the discovery of the tooth. The exact words are: Nella parte esterna del taglio (61) viene fuori (raccolto dalla S.na Tavanti) un dente che mi sembra umano (?) ma di dimensioni insolite (Neandertal?). La corona è piuttosto consunta. Le radici poderose, quadruplici forse fin quasi agli apici On the external part of the cut (61) a tooth appears (collected by Miss Tavanti) which looks human (?) but of unusual size (Neanderthal?). The crown is quite worn. The mighty roots, perhaps quadruplicated almost to the tips (Figure: Adriana Moroni).

**Figure S3:** 3D model of the crown (a, c) and enamel‐dentin junction (b, d) of GP1 in occlusal view, showing the crack affecting the enamel (a, dashed arrow) and dentin (b, dashed arrow), and the reconstructed morphology on the enamel (c, solid arrow) and dentin (d, solid arrow).

**Figure S4:** (a) Missing pseudo landmarks (red dots) on the crown outline. The four ones in line are located on the interproximal mesial facet, while the one on the opposite side lies on a cracked portion of the enamel, as detailed with the arrow in (b).

**Figure S5:** Reconstruction of the crown outline of GP1 based on the Neanderthal (a), early *H. sapiens* (b), Upper Paleolithic *H. sapiens* (c), recent *H. sapiens* (d), and overall mean (e).

**Figure S6:** Principal component analysis (PCA) plot of GP1 crown outlines reconstructed on the four comparative samples means and on the overall mean.

**Figure S7:** (Semi)landmark configuration for the 3D GM analysis of the UM1 in its occlusal (a) and buccal (b) view and the talus in its dorsal (c) and plantar (d) view.

**Figure S8:** Principal component analysis (PCA) plot of GP1 EDJ and CEJ reconstructed on the three comparative samples means and on the overall mean.

**Figure S9:** 3D digital model of the crown and enamel dentin‐junction of GP1 in occlusal (a, b), mesial (c, d) and disto‐buccal view (e, f), respectively. AC‐PAR = accessory ridge on the paracone.

**Figure S10:** Scatterplot between MD and BL diameters of GP1 and the comparative sample.

**Figure S11:** Principal component analysis (PCA) plot of the EDJ and CEJ of GP1 and the Neanderthal sample, with geographical data for the individuals. BE = Belgium; CR = Croatia; FR = France; GI = Gibraltar; IT = Italy; SP = Spain. As in Figure 6, the triangles are the comparative Neanderthal specimens, and the asterisk is GP1.

**Figure S12:** Principal component analysis (PCA) plot of the EDJ and CEJ of GP1 and the Neanderthal sample, with chronological data for the individuals. BE = Belgium; CR = Croatia; FR = France; GI = Gibraltar; IT = Italy; SP = Spain. As in Figure 6, the triangles are the comparative Neanderthal specimens, and the asterisk is GP1.

**Table S1:** List of the individuals used as comparative sample for MD and BL crown diameters analysis of GP1.
**Table S2:** List of the individuals used as comparative sample for 2D GM crown outline analysis of GP1.
**Table S3:** List of the individuals used as comparative sample for 3D GM EDJ and CEJ analysis of GP1.
**Table S4:** Data to perform the correlation analysis of the Neanderthal crown outlines with chronological age and geographic location.
**Table S5:** Data to perform the correlation analysis of the Neanderthal EDJs and CEJs with chronological age and geographic location.
**Table S6:** Results for permutation test for GP1 crown outline.
**Table S7:** Results of QDA and posterior probability for GP1 crown outline.
**Table S8:** Results of Pearson's product–moment correlation for GP1 crown outline.
**Table S9:** Results of permutation test for GP1 EDJ and CEJ.
**Table S10:** Results of LDA and posterior probability for GP1 EDJ and CEJ.
**Table S11:** Results of Pearson's product–moment correlation for GP1 EDJ and CEJ.

## Data Availability

All data supporting the conclusions of the GP1 study are available in the manuscript and/or in the Supporting Information. The newly generated 2D and 3D landmark and semilandmark coordinates for the GM analysis of GP1 are deposited in the AMS Acta Institutional Research Repository of the Alma Mater Studiorum—University of Bologna, accessible at the following link: https://doi.org/10.6092/unibo/amsacta/8337. For GP2, the 3D landmark and semilandmark coordinates of post‐industrial recent *H. sapiens* tali used as comparison are available from https://doi.org/10.1371/journal.pone.0229255. The 3D landmark and semilandmark coordinates of the remaining comparative tali can be obtained from the corresponding author RS upon request.
