# Supplementary figures and images for: The Human Remains From the MIS 6 Site of Grotta Del Poggio (Cilento, Southern Italy): A Taxonomic and Chronological Reassessment
Source: Am J Biol Anthropol. 2025 Dec 28;188(4):e70188. doi: 10.1002/ajpa.70188 (PMC12745066; doi:10.1002/ajpa.70188)

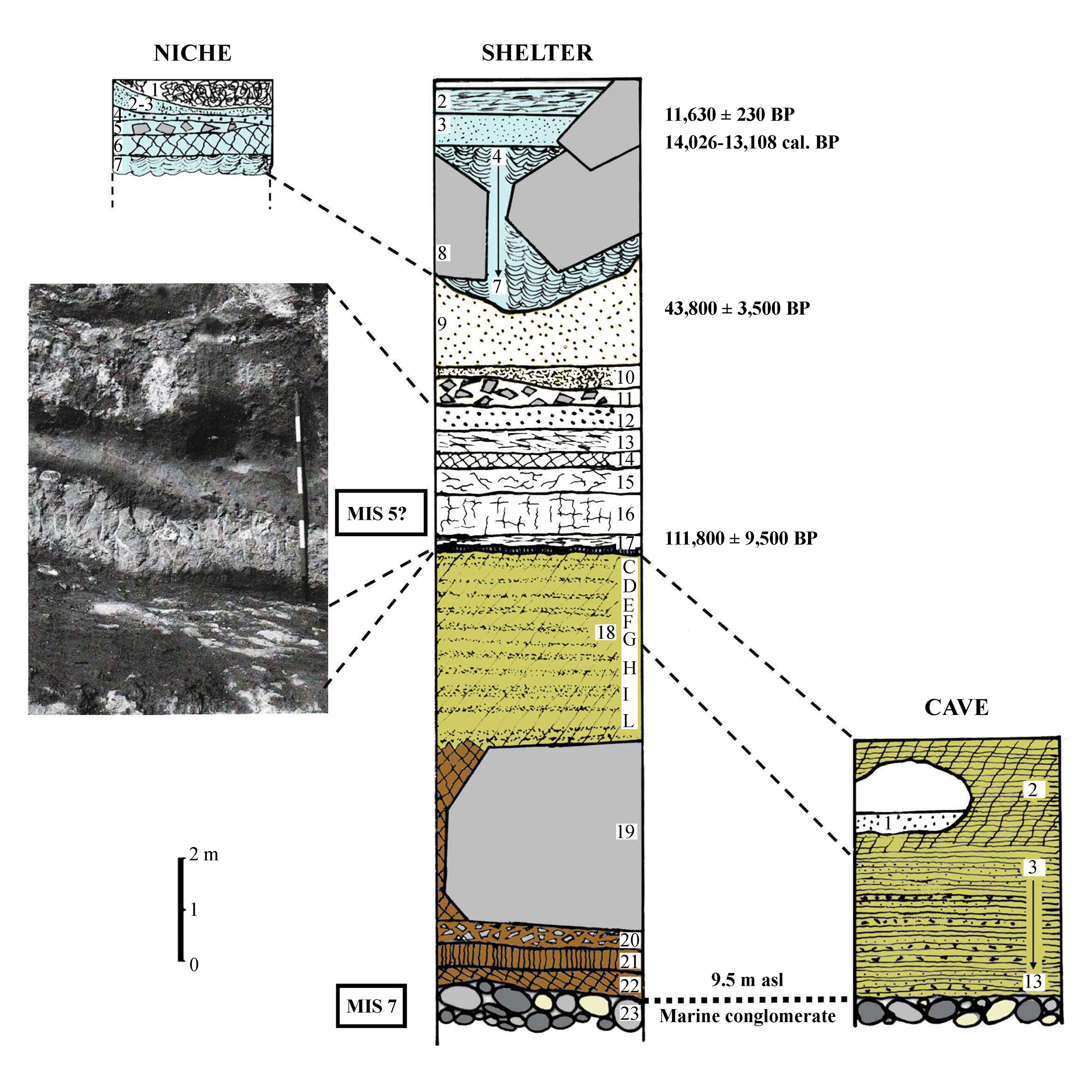

Supplement: Supplementary file 1 — Figure S1: Poggio. Stratigraphic correlations between Cave, shelter and niche. On the left: stratigraphic detail of layers 18 to 12 of the shelter in which the top surface of layer 18 can be seen sealed by a stalagmitic crust tentatively referred to MIS5e on the basis of a TL date (111.800 ± 9.500) obtained in the following layer 17 (from Gambassini's archive modified). The series of Poggio lies on a marine conglomerate 9.5 m asl attributed to MIS7, on geological and biostratigraphic grounds. Layers 18 of the shelter and layers 13–2 of the cave contain pre‐Levallois industries, whilst the lithic assemblages of layers 17 to 9 of the shelter are characterized by the employment of the Levallois method. Layers 7 to 2 of the shelter have yielded Epigravettian industries (Boscato et al. 2009). [file AJPA-188-e70188-s002.tif]

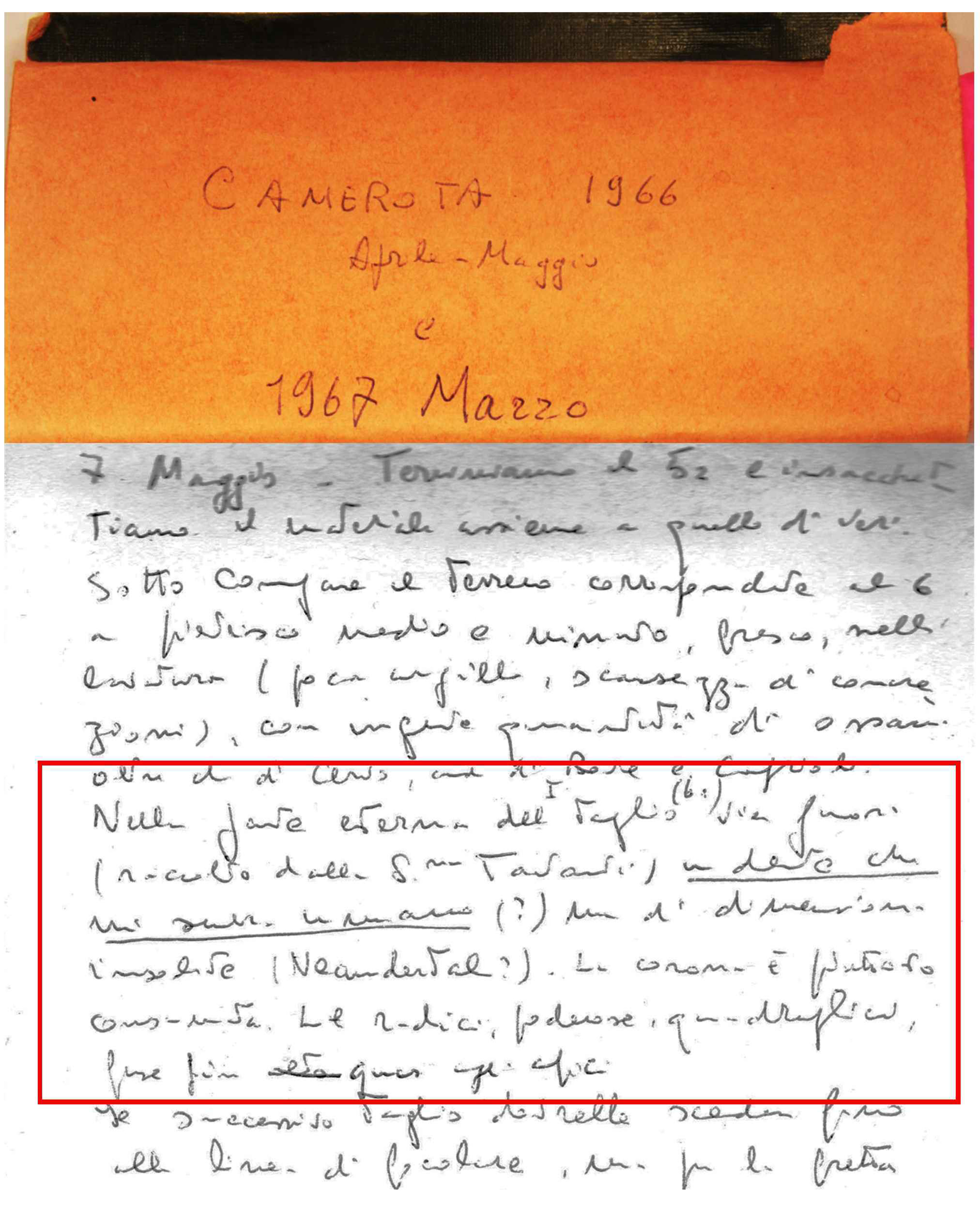

Supplement: Supplementary file 2 — Figure S2: Excerpt from Palma di Cesnola's excavation field notes, dated May 7th, 1966, documenting the discovery of the tooth. The exact words are: Nella parte esterna del taglio (61) viene fuori (raccolto dalla S.na Tavanti) un dente che mi sembra umano (?) ma di dimensioni insolite (Neandertal?). La corona è piuttosto consunta. Le radici poderose, quadruplici forse fin quasi agli apici On the external part of the cut (61) a tooth appears (collected by Miss Tavanti) which looks human (?) but of unusual size (Neanderthal?). The crown is quite worn. The mighty roots, perhaps quadruplicated almost to the tips (Figure: Adriana Moroni). [file AJPA-188-e70188-s004.tif]

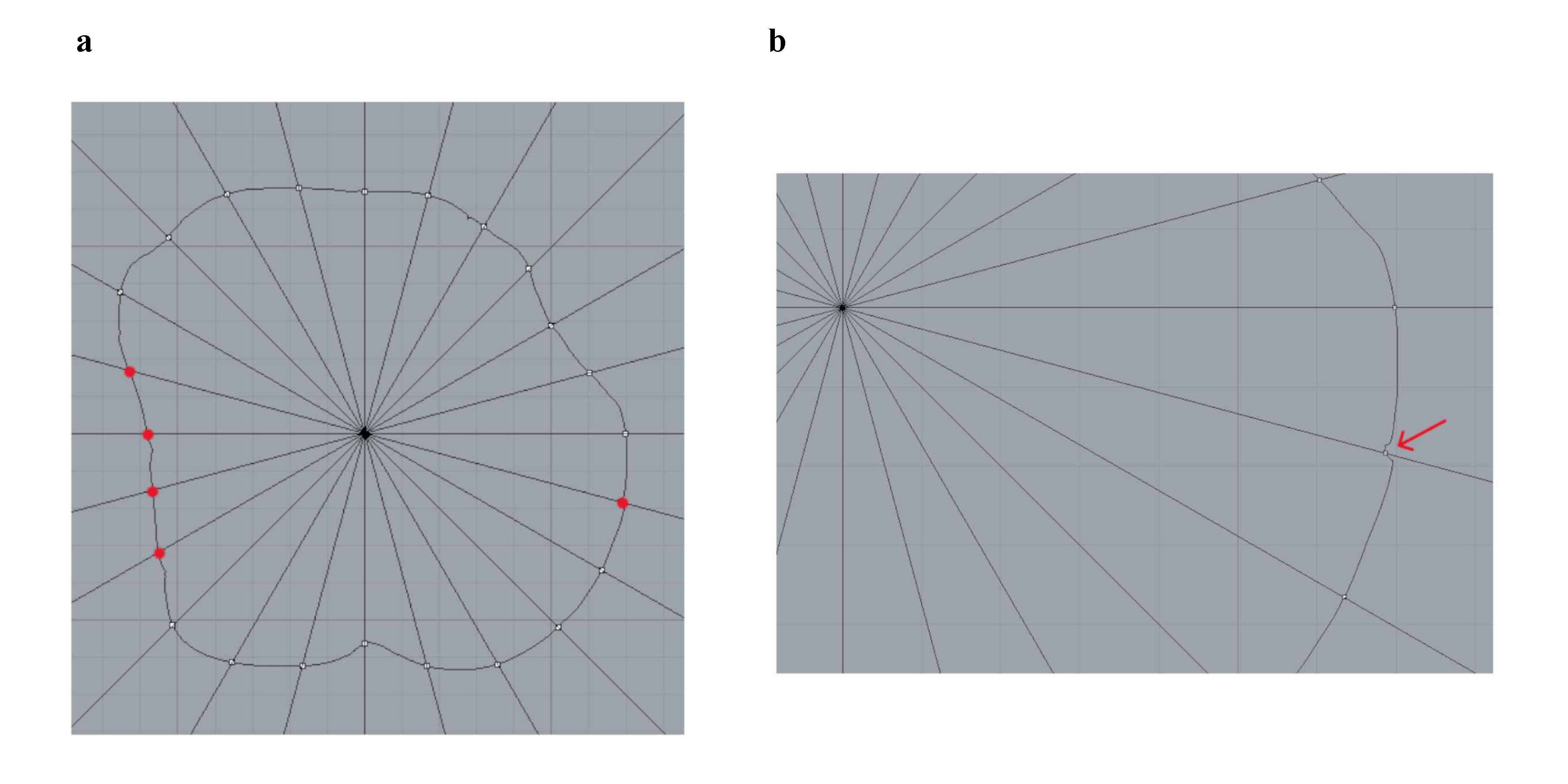

Supplement: Supplementary file 3 — Figure S3: 3D model of the crown (a, c) and enamel‐dentin junction (b, d) of GP1 in occlusal view, showing the crack affecting the enamel (a, dashed arrow) and dentin (b, dashed arrow), and the reconstructed morphology on the enamel (c, solid arrow) and dentin (d, solid arrow). [file AJPA-188-e70188-s012.zip › ajpa70188-sup-0006-FigureS3@Fig_S3_2.tif]

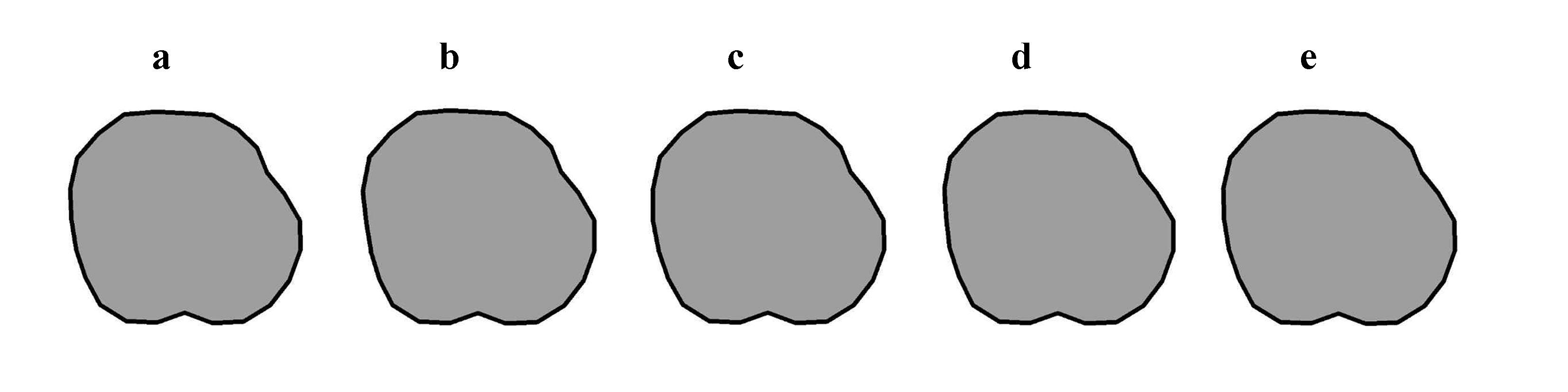

Supplement: Supplementary file 4 — Figure S4: (a) Missing pseudo landmarks (red dots) on the crown outline. The four ones in line are located on the interproximal mesial facet, while the one on the opposite side lies on a cracked portion of the enamel, as detailed with the arrow in (b). [file AJPA-188-e70188-s009.zip › ajpa70188-sup-0008-FigureS4@Fig_S4_2.tif]

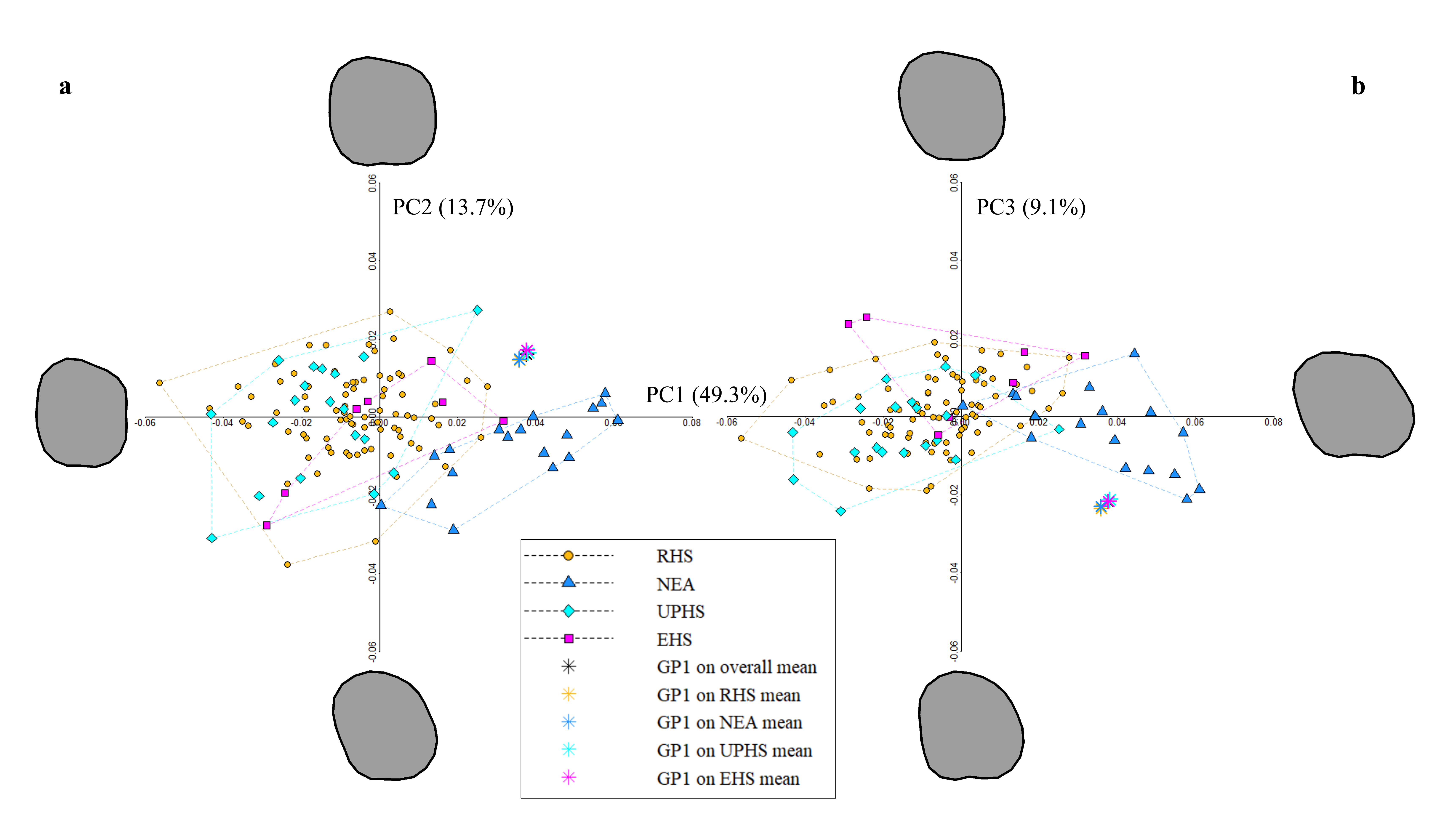

Supplement: Supplementary file 5 — Figure S5: Reconstruction of the crown outline of GP1 based on the Neanderthal (a), early H. sapiens (b), Upper Paleolithic H. sapiens (c), recent H. sapiens (d), and overall mean (e). [file AJPA-188-e70188-s001.zip › ajpa70188-sup-0010-FigureS5@Fig_S5_2.tif]

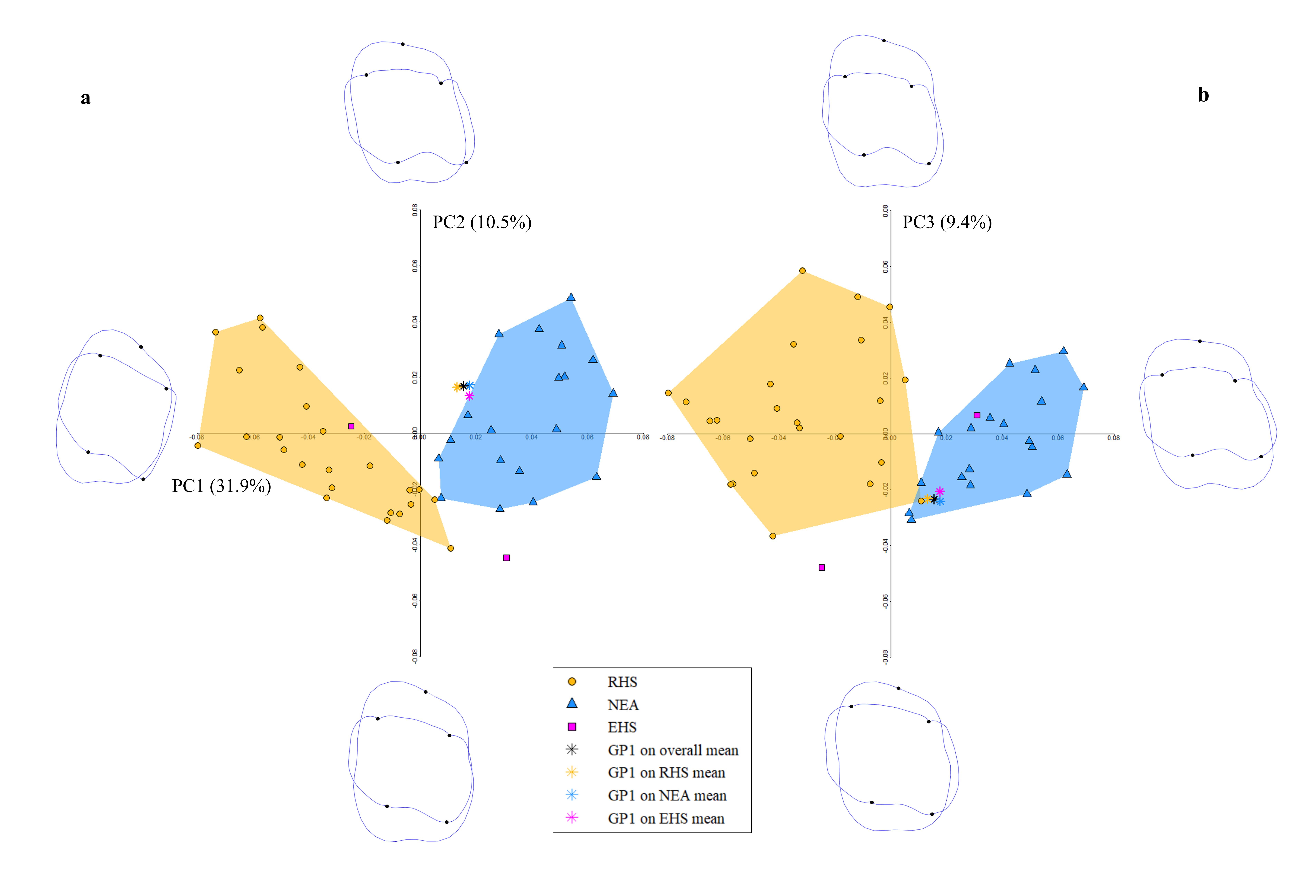

Supplement: Supplementary file 6 — Figure S6: Principal component analysis (PCA) plot of GP1 crown outlines reconstructed on the four comparative samples means and on the overall mean. [file AJPA-188-e70188-s003.zip › ajpa70188-sup-0012-FigureS6@Fig_S6_2.tif]

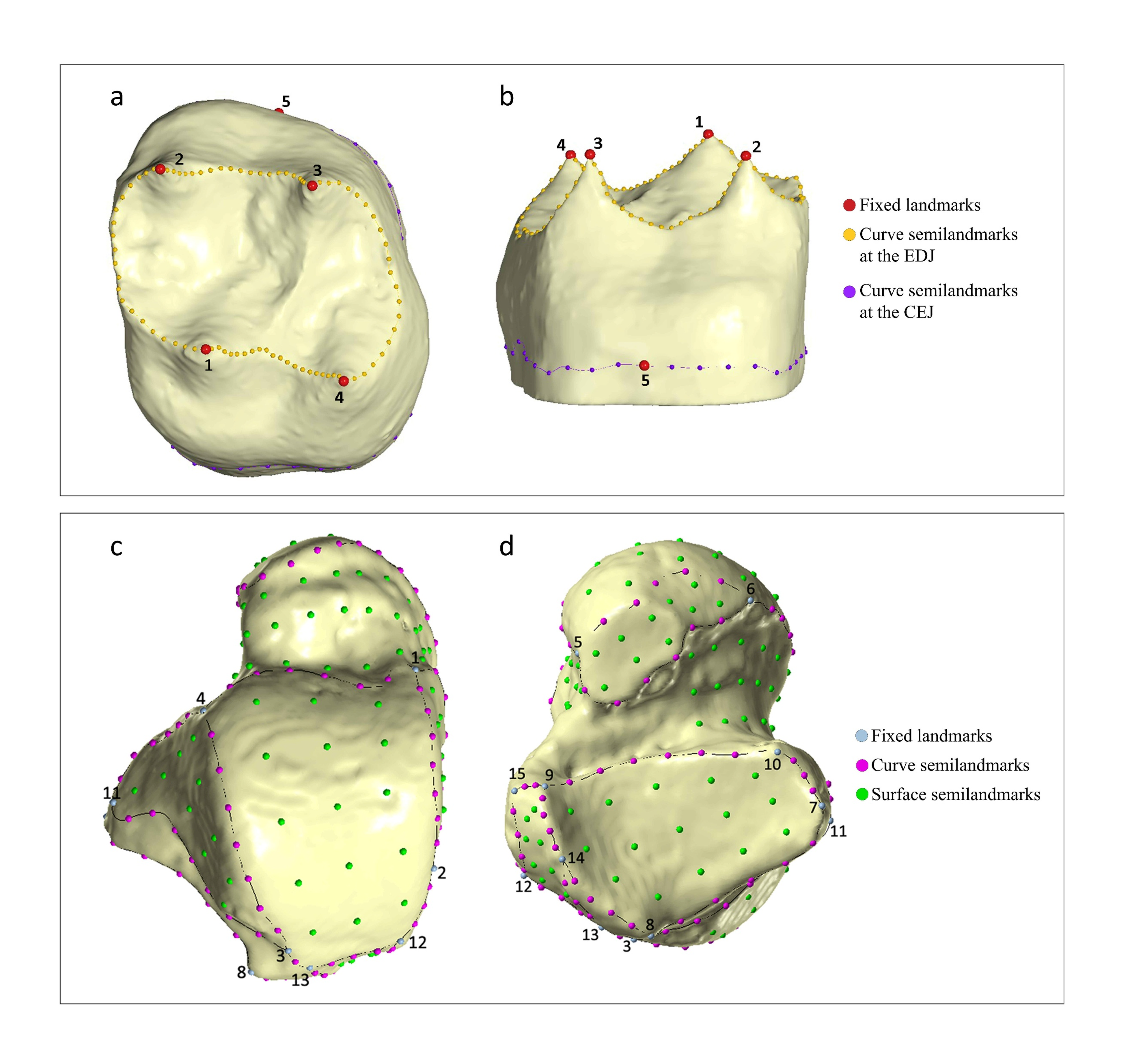

Supplement: Supplementary file 7 — Figure S7: (Semi)landmark configuration for the 3D GM analysis of the UM1 in its occlusal (a) and buccal (b) view and the talus in its dorsal (c) and plantar (d) view. [file AJPA-188-e70188-s005.zip › ajpa70188-sup-0015-FigureS7@Fig_S7.tiff]

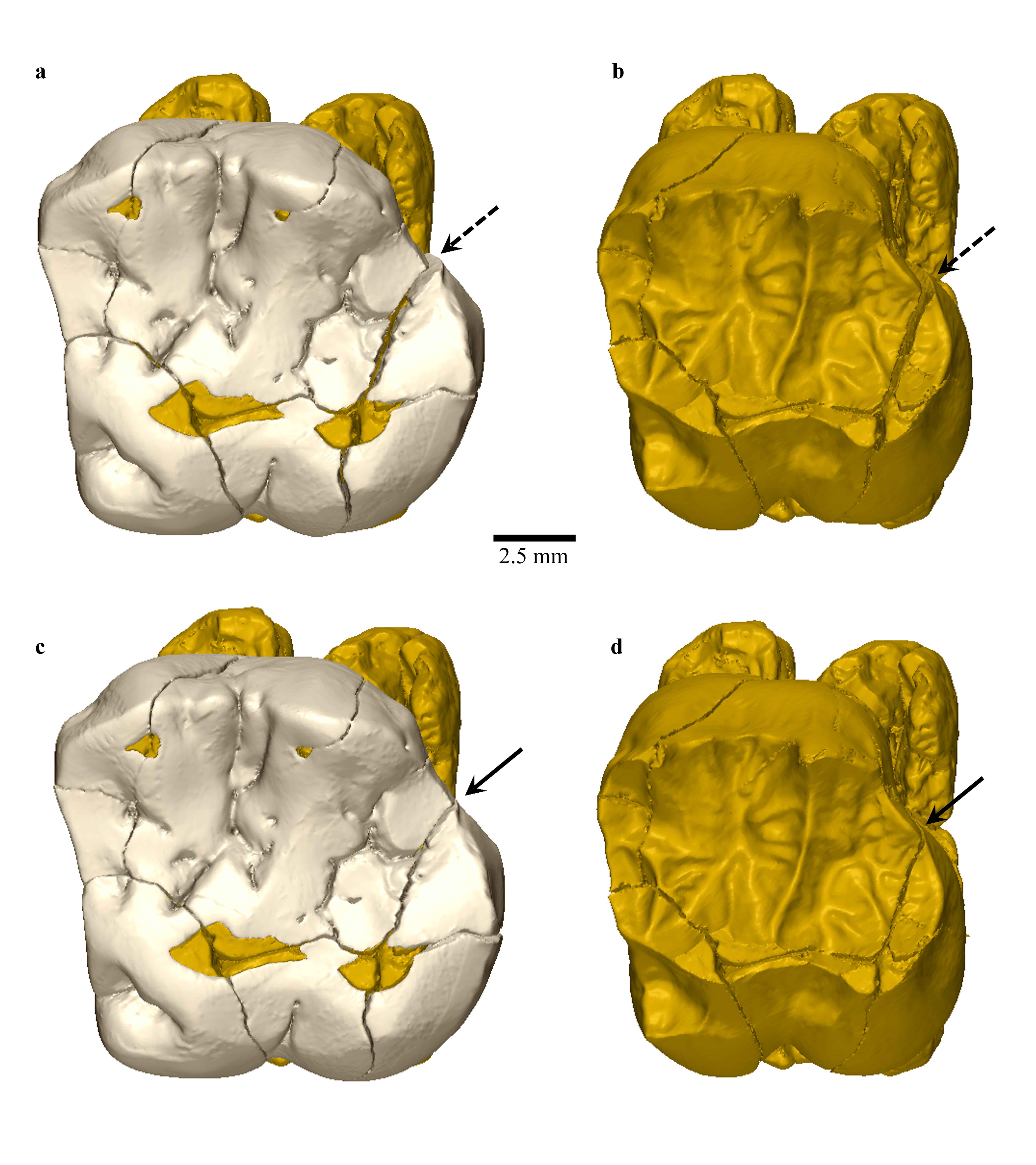

Supplement: Supplementary file 7 — Figure S7: (Semi)landmark configuration for the 3D GM analysis of the UM1 in its occlusal (a) and buccal (b) view and the talus in its dorsal (c) and plantar (d) view. [file AJPA-188-e70188-s005.zip › ajpa70188-sup-0014-FigureS7@Fig_S7.tif]

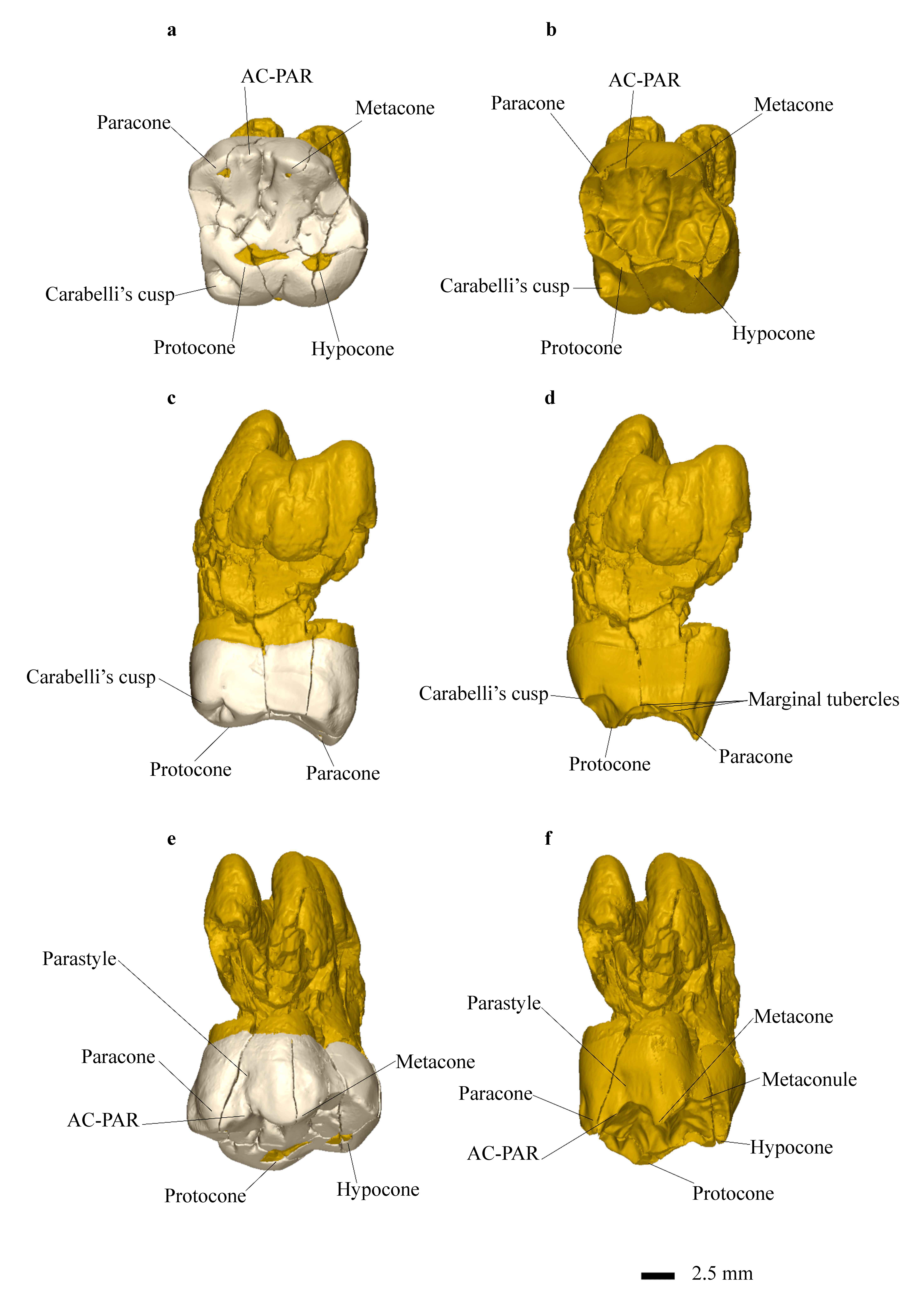

Supplement: Supplementary file 8 — Figure S8: Principal component analysis (PCA) plot of GP1 EDJ and CEJ reconstructed on the three comparative samples means and on the overall mean. [file AJPA-188-e70188-s008.zip › ajpa70188-sup-0017-FigureS8@Fig_S8_2.tif]

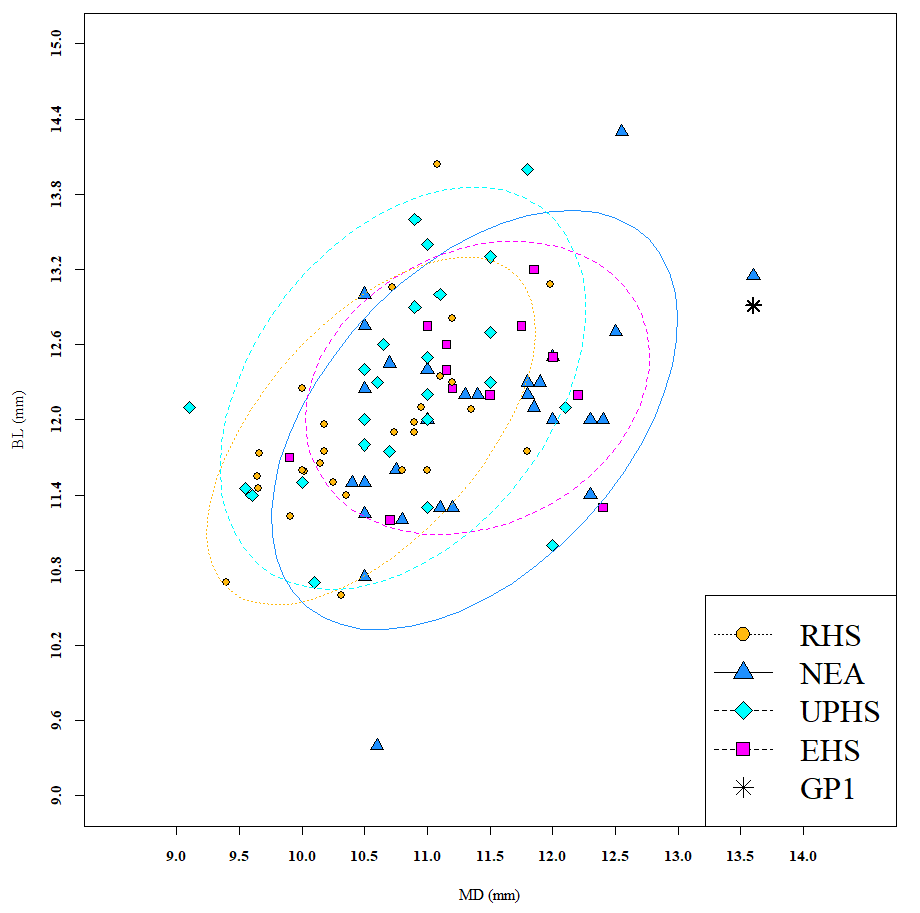

Supplement: Supplementary file 9 — Figure S9: 3D digital model of the crown and enamel dentin‐junction of GP1 in occlusal (a, b), mesial (c, d) and disto‐buccal view (e, f), respectively. AC‐PAR = accessory ridge on the paracone. [file AJPA-188-e70188-s013.zip › ajpa70188-sup-0018-FigureS9@Fig_S9.tiff]

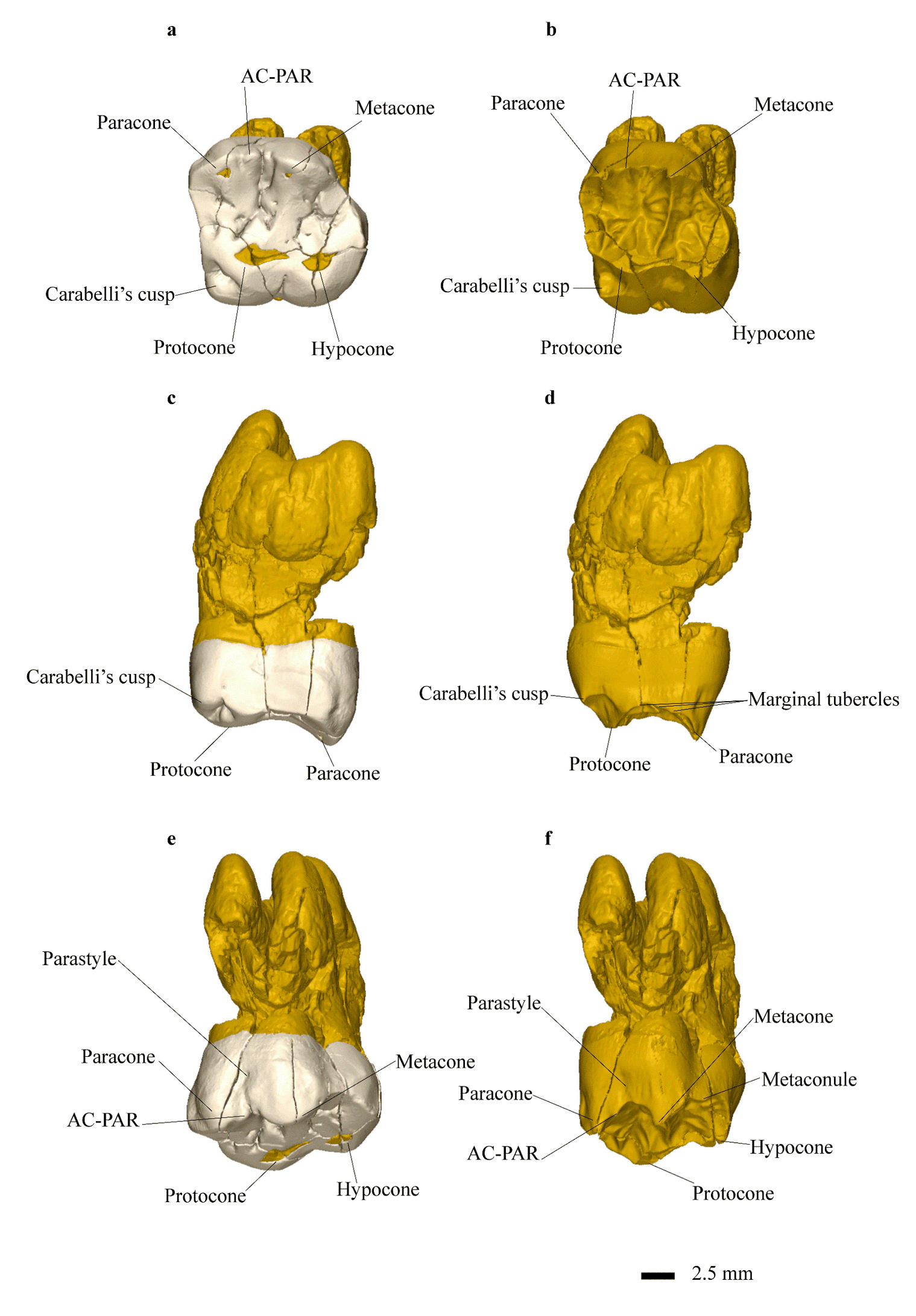

Supplement: Supplementary file 9 — Figure S9: 3D digital model of the crown and enamel dentin‐junction of GP1 in occlusal (a, b), mesial (c, d) and disto‐buccal view (e, f), respectively. AC‐PAR = accessory ridge on the paracone. [file AJPA-188-e70188-s013.zip › ajpa70188-sup-0019-FigureS9@Fig_S9_1.tif]

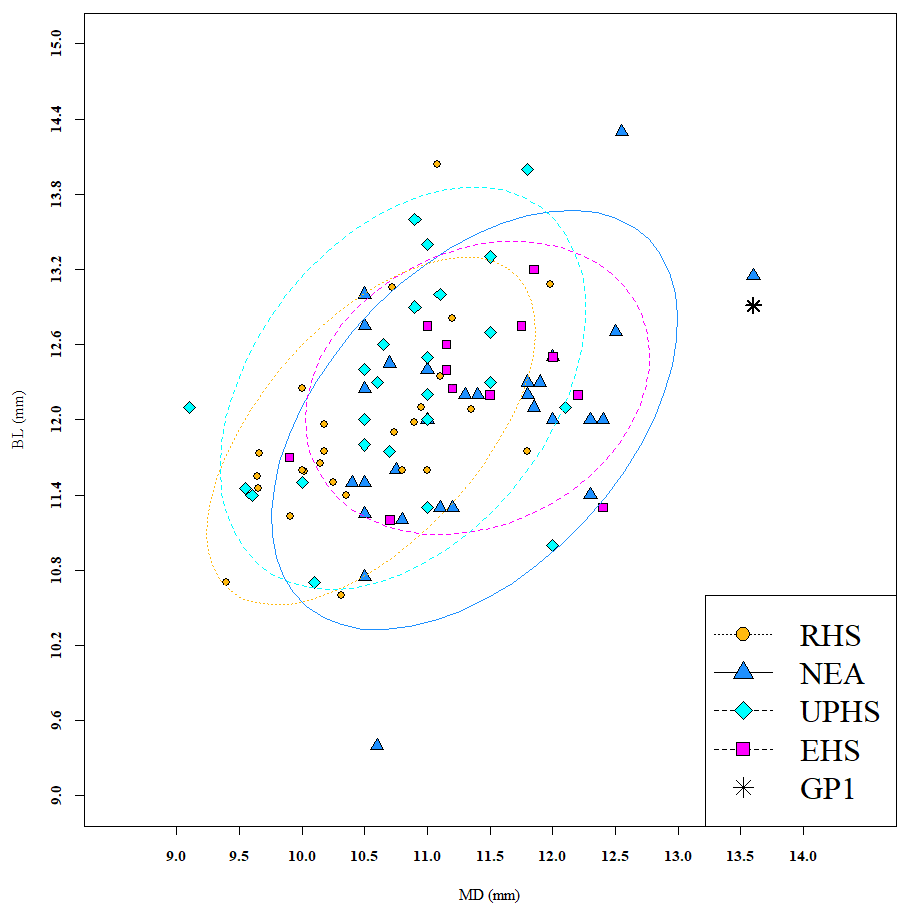

Supplement: Supplementary file 10 — Figure S10: Scatterplot between MD and BL diameters of GP1 and the comparative sample. [file AJPA-188-e70188-s007.tif]

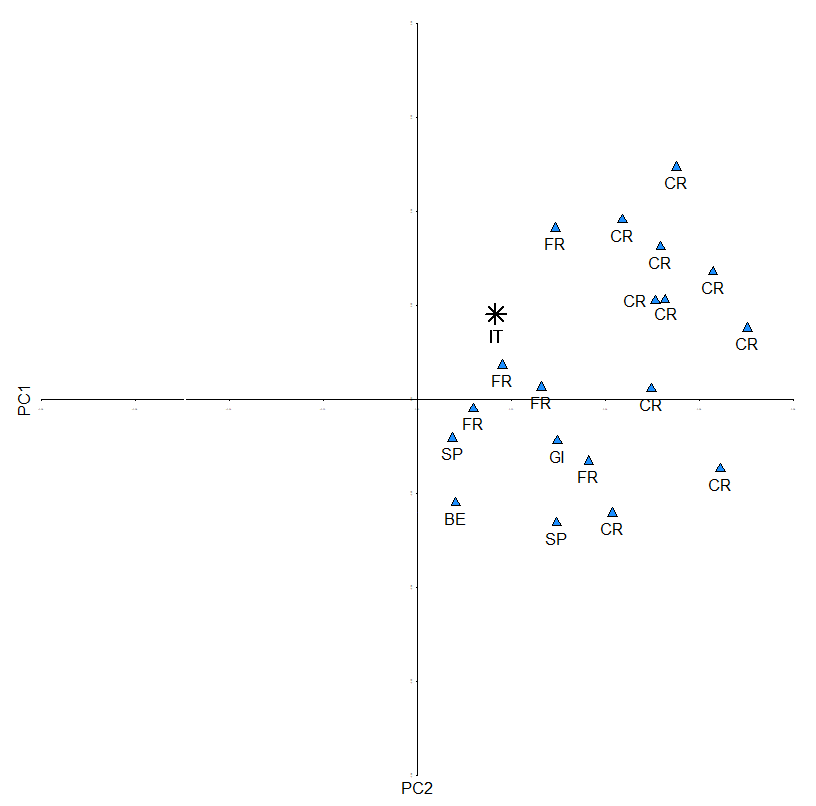

Supplement: Supplementary file 11 — Figure S11: Principal component analysis (PCA) plot of the EDJ and CEJ of GP1 and the Neanderthal sample, with geographical data for the individuals. BE = Belgium; CR = Croatia; FR = France; GI = Gibraltar; IT = Italy; SP = Spain. As in Figure 6, the triangles are the comparative Neanderthal specimens, and the asterisk is GP1. [file AJPA-188-e70188-s006.tif]

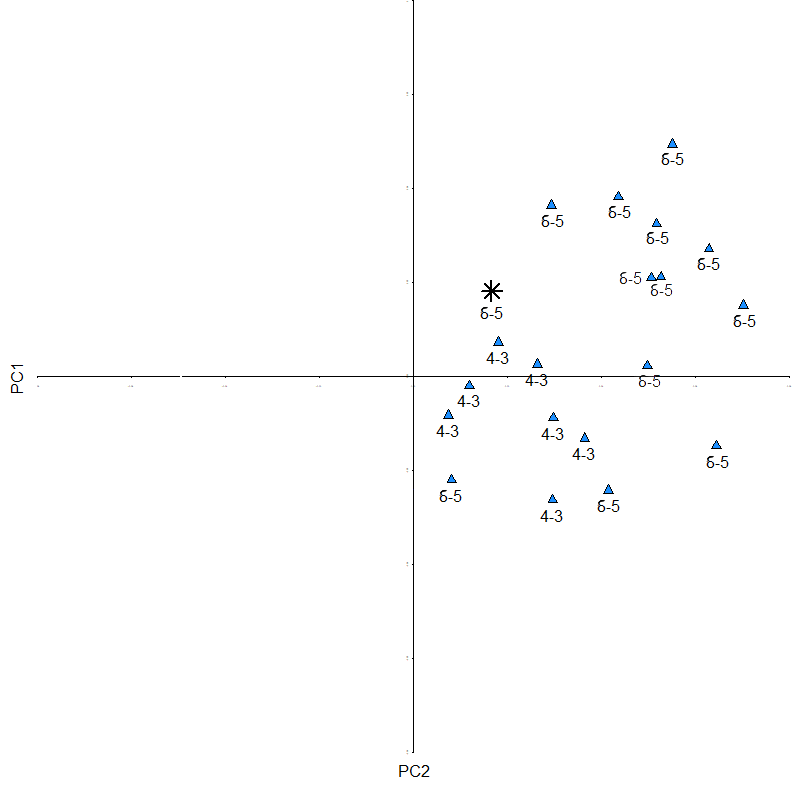

Supplement: Supplementary file 12 — Figure S12: Principal component analysis (PCA) plot of the EDJ and CEJ of GP1 and the Neanderthal sample, with chronological data for the individuals. BE = Belgium; CR = Croatia; FR = France; GI = Gibraltar; IT = Italy; SP = Spain. As in Figure 6, the triangles are the comparative Neanderthal specimens, and the asterisk is GP1. [file AJPA-188-e70188-s011.tif]
