# Supplementary material for: The Human Remains From the MIS 6 Site of Grotta Del Poggio (Cilento, Southern Italy): A Taxonomic and Chronological Reassessment
Source: Am J Biol Anthropol. 2025 Dec 28;188(4):e70188. doi: 10.1002/ajpa.70188 (PMC12745066; doi:10.1002/ajpa.70188)
Supplement: Supplementary file 13 — Table S1: List of the individuals used as comparative sample for MD and BL crown diameters analysis of GP1. Table S2: List of the individuals used as comparative sample for 2D GM crown outline analysis of GP1. Table S3: List of the individuals used as comparative sample for 3D GM EDJ and CEJ analysis of GP1. Table S4: Data to perform the correlation analysis of the Neanderthal crown outlines with chronological age and geographic location. Table S5: Data to perform the correlation analysis of the Neanderthal EDJs and CEJs with chronological age and geographic location. Table S6: Results for permutation test for GP1 crown outline. Table S7: Results of QDA and posterior probability for GP1 crown outline. Table S8: Results of Pearson's product–moment correlation for GP1 crown outline. Table S9: Results of permutation test for GP1 EDJ and CEJ. Table S10: Results of LDA and posterior probability for GP1 EDJ and CEJ. Table S11: Results of Pearson's product–moment correlation for GP1 EDJ and CEJ. [file AJPA-188-e70188-s010.docx]

Table S1

List of the individuals used as comparative sample for MD and BL crown diameters analysis of GP1.

| Specimen | Comparative group | MD | BL | Source of the crown diameters |
| --- | --- | --- | --- | --- |
| Dogee Bary K16 SK 1 | RHS | 9.4 | 10.7 | Benazzi et al. (2011b) |
| Bystrovka 2 K9 N15 a | RHS | 10 | 12.25 | Benazzi et al. (2011b) |
| Kopto K4 M3b | RHS | 10.9 | 11.98 | Benazzi et al. (2011b) |
| Bystrovka 2 K4 N20 a | RHS | 11 | 11.6 | Benazzi et al. (2011b) |
| Bystrovka 3 K6 M6 | RHS | 11.8 | 11.75 | Benazzi et al. (2011b) |
| La Rochette 3 | UPHS | 9.1 | 12.1 | Frayer (1978) |
| Kostienki XIV [Markina Gora] 2 | UPHS | 9.55 | 11.45 | Benazzi et al. (2011b) |
| Predmosti 4 | UPHS | 9.6 | 11.4 | Frayer (1978) |
| Bruckneudorf G899 | RHS | 9.64 | 11.55 | Benazzi et al. (2011b) |
| NHMW 15358 | RHS | 9.66 | 11.73 | Benazzi et al. (2011b) |
| Skhul 2 | EHS | 9.9 | 11.7 | Frayer (1978) |
| NHMW 6034 | RHS | 9.91 | 11.23 | Benazzi et al. (2011b) |
| Predmosti 10 | UPHS | 10 | 11.5 | Frayer (1978) |
| NHMW 15357 | RHS | 10.02 | 11.59 | Benazzi et al. (2011b) |
| Kostenki 3 | UPHS | 10.1 | 10.7 | Benazzi et al. (2011b) |
| NHMW 6031 | RHS | 10.18 | 11.96 | Benazzi et al. (2011b) |
| Bruckneudorf G905/1 | RHS | 10.31 | 10.6 | Benazzi et al. (2011b) |
| Spy 1 | NEA | 10.4 | 11.5 | Frayer (1978) |
| Châteauneuf 2 | NEA | 10.5 | 10.75 | Frayer (1978) |
| Saccopastore 2 | NEA | 10.5 | 11.25 | Frayer (1978) |
| Tabun 901 | NEA | 10.5 | 11.5 | Frayer (1978) |
| Lagar Velho 1 | UPHS | 10.5 | 11.8 | Hillson and Trinkaus (2002) |
| Combe Capelle 1 | UPHS | 10.5 | 12 | Frayer (1978) |
| Shanidar 2 | NEA | 10.5 | 12.25 | Trinkaus (1983) |
| Predmosti 7 | UPHS | 10.5 | 12.4 | Frayer (1978) |
| La Quina 5 | NEA | 10.5 | 12.75 | Frayer (1978) |
| La Ferrassie 2 | NEA | 10.5 | 13 | Frayer (1978) |
| Gibraltar 917 | NEA | 10.6 | 9.4 | Frayer (1978) |
| Mladec 908 | UPHS | 10.6 | 12.3 | Teschler-Nicola (2006) |
| Kostenki 4 | UPHS | 10.65 | 12.6 | Benazzi et al. (2011b) |
| Skhul 7 | EHS | 10.7 | 11.2 | Frayer (1978) |
| Sungir 3 | UPHS | 10.7 | 11.75 | Benazzi et al. (2011b) |
| Amud 1 | NEA | 10.7 | 12.45 | Suzuki and Takai (1970) |
| NHMW 811 | RHS | 10.72 | 13.06 | Benazzi et al. (2011b) |
| Tabun 1 | NEA | 10.75 | 11.6 | Frayer (1978) |
| La Croze de Dua 1 | NEA | 10.8 | 11.2 | Frayer (1978) |
| Fontechevade 2 | UPHS | 10.9 | 12.9 | Frayer (1978) |
| Mladec 907 | UPHS | 10.9 | 13.6 | Teschler-Nicola (2006) |
| Ushauz Cave Sk 1 | RHS | 10.95 | 12.1 | Benazzi et al. (2011b) |
| Predmosti 1 | UPHS | 11 | 11.3 | Frayer (1978) |
| Shanidar 4 | NEA | 11 | 12 | Trinkaus (1983) |
| Predmosti 5 | UPHS | 11 | 12 | Frayer (1978) |
| Monsempron 4 | NEA | 11 | 12 | Frayer (1978) |
| Predmosti 2 | UPHS | 11 | 12.2 | Frayer (1978) |
| Jersey 1 | NEA | 11 | 12.4 | Frayer (1978) |
| Mladec 901 | UPHS | 11 | 12.5 | Teschler-Nicola (2006) |
| Qafzeh 6 | EHS | 11 | 12.75 | Vandermeersch (1981) |
| Sungir 2 | UPHS | 11 | 13.4 | Benazzi et al. (2011b) |
| NHMW Breitinger Nr. 85 | RHS | 11.08 | 14.04 | Benazzi et al. (2011b) |
| Kulna Cave 1 | NEA | 11.1 | 11.3 | Benazzi et al. (2011b) |
| Mladec 905 | UPHS | 11.1 | 13 | Teschler-Nicola (2006) |
| Skhul 4 | EHS | 11.15 | 12.4 | Frayer (1978) |
| Skhul 5 | EHS | 11.15 | 12.6 | Frayer (1978) |
| NHMW Breitinger Nr. 87 | RHS | 11.2 | 12.81 | Benazzi et al. (2011b) |
| Tabun 902 | NEA | 11.2 | 11.3 | Frayer (1978) |
| Qafzeh 4 | EHS | 11.2 | 12.25 | Vandermeersch (1981) |
| Saccopastore 1 | NEA | 11.3 | 12.2 | Frayer (1978) |
| NHMW 15353 | RHS | 11.35 | 12.08 | Benazzi et al. (2011b) |
| Spy 2 | NEA | 11.4 | 12.2 | Frayer (1978) |
| Skhul 6 | EHS | 11.5 | 12.2 | Frayer (1978) |
| Predmosti 14 | UPHS | 11.5 | 12.3 | Frayer (1978) |
| Grotte des Enfants 6 | UPHS | 11.5 | 12.7 | Frayer (1978) |
| Predmosti 3 | UPHS | 11.5 | 13.3 | Frayer (1978) |
| Qafzeh 7 | EHS | 11.75 | 12.75 | Vandermeersch (1981) |
| Tabun 903 | NEA | 11.8 | 12.3 | Frayer (1978) |
| Cro-Magnon 5 | UPHS | 11.8 | 14 | Frayer (1978) |
| Shanidar 1 | NEA | 11.85 | 12.1 | Trinkaus (1983) |
| Qafzeh 9 | EHS | 11.85 | 13.2 | Vandermeersch (1981) |
| KRP Maxilla D | NEA | 11.9 | 12.3 | Wolpoff (1979) |
| NHMW 9687 | RHS | 11.98 | 13.08 | Benazzi et al. (2011b) |
| Ksar Akil 1 | UPHS | 12 | 11 | Frayer (1978) |
| La Quina 18 | NEA | 12 | 12 | Frayer (1978) |
| Le Moustier 1 | NEA | 12 | 12.5 | Frayer (1978) |
| Skhul 11 | EHS | 12 | 12.5 | Frayer (1978) |
| Predmosti 9 | UPHS | 12.1 | 12.1 | Frayer (1978) |
| Skhul 9 | EHS | 12.2 | 12.2 | Frayer (1978) |
| Starosel'e 1 | NEA | 12.3 | 11.4 | Frayer (1978) |
| La Quina 13 | NEA | 12.3 | 12 | Frayer (1978) |
| Skhul 1 | EHS | 12.4 | 11.3 | Frayer (1978) |
| KRP Maxilla B | NEA | 12.4 | 12 | Wolpoff (1979) |
| Teshik-Tash 1 | NEA | 12.5 | 12.7 | Shpakova (2001) |
| KRP Maxilla C | NEA | 13.6 | 13.15 | Wolpoff (1979) |
| Bystrovka 2 K3 N8 | RHS | 10.35 | 11.4 | Benazzi et al. (2011b) |
| Bystrovka 3 K7 N39 | RHS | 9.65 | 11.45 | Benazzi et al. (2011b) |
| Bystrovka 3 K6 M11 | RHS | 10.25 | 11.5 | Benazzi et al. (2011b) |
| Bystrovka 3 K7 N4 | RHS | 10 | 11.6 | Benazzi et al. (2011b) |
| Bystrovka 3 K7 N 30 | RHS | 10.8 | 11.6 | Benazzi et al. (2011b) |
| Bystrovka 3 K6 N9 | RHS | 10.15 | 11.65 | Benazzi et al. (2011b) |
| Kopto K4 M3a | RHS | 10.18 | 11.75 | Benazzi et al. (2011b) |
| Kopto K4 M1a | RHS | 10.74 | 11.9 | Benazzi et al. (2011b) |
| Bystrovka 2 K3 n14 | RHS | 11.1 | 12.35 | Benazzi et al. (2011b) |
| Obi Rakhmat 1 | NEA | 12.55 | 14.3 | Benazzi et al. (2011b) |
| Taddeo | NEA | 11.8 | 12.2 | Benazzi et al. (2011b) |
| GP1 | - | 13.6 | 12.91 | This work |

Table S2

List of the individuals used as comparative sample for 2D GM crown outline analysis of GP1.

| Specimen | Comparative group | Reference | Source of the coordinates |
| --- | --- | --- | --- |
| IND 6 9773 | RHS | Bailey et al. (2014) | This work |
| IND 58 6 24 154 | RHS | Bailey et al. (2014) | This work |
| NAM AK 99 1 193 | RHS | Bailey et al. (2014) | This work |
| NAM AK 99 1 83 | RHS | Bailey et al. (2014) | This work |
| NAM AZ 99 8292 | RHS | Bailey et al. (2014) | This work |
| NAM AZ 99 9154 | RHS | Bailey et al. (2014) | This work |
| SAF A1244 | RHS | Bailey et al. (2014) | This work |
| SAF A1320 | RHS | Bailey et al. (2014) | This work |
| SAF A1439 | RHS | Bailey et al. (2014) | This work |
| SAF A3148 | RHS | Bailey et al. (2014) | This work |
| SAF UCT 51 | RHS | Bailey et al. (2014) | This work |
| SAF UCT 58C | RHS | Bailey et al. (2014) | This work |
| SAF UCT 231 | RHS | Bailey et al. (2014) | This work |
| SAF UCT 468 | RHS | Bailey et al. (2014) | This work |
| SAF UCT 471 | RHS | Bailey et al. (2014) | This work |
| BRIT COL 99 1622 | RHS | Bailey et al. (2014) | This work |
| BRIT COL 99 4551 | RHS | Bailey et al. (2014) | This work |
| BRIT COL 99 1658 | RHS | Bailey et al. (2014) | This work |
| BRIT COL 99 4657 | RHS | Bailey et al. (2014) | This work |
| HUNGARY VL 4411 | RHS | Bailey et al. (2014) | This work |
| HUNGARY VL 4970 | RHS | Bailey et al. (2014) | This work |
| MEXICO 99 9629 | RHS | Bailey et al. (2014) | This work |
| PB 23 | RHS | Bailey et al. (2014) | This work |
| PB 225 | RHS | Bailey et al. (2014) | This work |
| PB 227 | RHS | Bailey et al. (2014) | This work |
| PB 246 | RHS | Bailey et al. (2014) | This work |
| PB 292 | RHS | Bailey et al. (2014) | This work |
| PB 294 | RHS | Bailey et al. (2014) | This work |
| PB 321 | RHS | Bailey et al. (2014) | This work |
| PB 335B | RHS | Bailey et al. (2014) | This work |
| PB 379 | RHS | Bailey et al. (2014) | This work |
| PB 414 | RHS | Bailey et al. (2014) | This work |
| PB 419 | RHS | Bailey et al. (2014) | This work |
| PB 452 | RHS | Bailey et al. (2014) | This work |
| PB 464 | RHS | Bailey et al. (2014) | This work |
| PB 492 | RHS | Bailey et al. (2014) | This work |
| PB 544 | RHS | Bailey et al. (2014) | This work |
| PB 601 | RHS | Bailey et al. (2014) | This work |
| PB 636 | RHS | Bailey et al. (2014) | This work |
| PB 674 | RHS | Bailey et al. (2014) | This work |
| PB 868 | RHS | Bailey et al. (2014) | This work |
| PB 891 | RHS | Bailey et al. (2014) | This work |
| PB 940 | RHS | Bailey et al. (2014) | This work |
| PB 1009 | RHS | Bailey et al. (2014) | This work |
| PB 1010 | RHS | Bailey et al. (2014) | This work |
| PB 1132 | RHS | Bailey et al. (2014) | This work |
| PB 1255 | RHS | Bailey et al. (2014) | This work |
| PERU 99 1 876 | RHS | Bailey et al. (2014) | This work |
| PERU 99 1892A | RHS | Bailey et al. (2014) | This work |
| PERU 2765 | RHS | Bailey et al. (2014) | This work |
| PERU VL 190 | RHS | Bailey et al. (2014) | This work |
| PERU VL 559 | RHS | Bailey et al. (2014) | This work |
| PERU VL 787 | RHS | Bailey et al. (2014) | This work |
| PERU VL 2763 | RHS | Bailey et al. (2014) | This work |
| PERU VL 2766 | RHS | Bailey et al. (2014) | This work |
| Arizona AMNH 99 9175 | RHS | Bailey et al. (2014) | This work |
| Asia Minor AMNH VL 7229 | RHS | Bailey et al. (2014) | This work |
| Austria AMNH VL 1357 | RHS | Bailey et al. (2014) | This work |
| Austria AMNH VL 3677 | RHS | Bailey et al. (2014) | This work |
| Bangkok AMNG VL 2448 | RHS | Bailey et al. (2014) | This work |
| Bangkok AMNH VL 2450 | RHS | Bailey et al. (2014) | This work |
| British Columbia AMNH 99 2619 | RHS | Bailey et al. (2014) | This work |
| British Columbia AMNH 99 3645 | RHS | Bailey et al. (2014) | This work |
| Germany AMNH VL 2770 2779 | RHS | Bailey et al. (2014) | This work |
| Greece AMNH VL 2192 | RHS | Bailey et al. (2014) | This work |
| Hungary AMNH VL 2557 | RHS | Bailey et al. (2014) | This work |
| Hungary AMNH VL 2622 | RHS | Bailey et al. (2014) | This work |
| IT 1105 | RHS | Bailey et al. (2014) | This work |
| Marquesas Islands AMNH 99 1 1511 | RHS | Bailey et al. (2014) | This work |
| Marquesas Islands AMNH 99 1 1991 | RHS | Bailey et al. (2014) | This work |
| Mexico AMNH 99 144 | RHS | Bailey et al. (2014) | This work |
| Mexico AMNH 99 9711 | RHS | Bailey et al. (2014) | This work |
| PB9 | RHS | Bailey et al. (2014) | This work |
| PB149 | RHS | Bailey et al. (2014) | This work |
| Peru AMNH 1 1076 | RHS | Bailey et al. (2014) | This work |
| Peru AMNH B99 3698 | RHS | Bailey et al. (2014) | This work |
| Peru AMNH VL 2759 | RHS | Bailey et al. (2014) | This work |
| Turkey AMNH VL 1015 | RHS | Bailey et al. (2014) | This work |
| Utah AMNH 99 7430 | RHS | Bailey et al. (2014) | This work |
| West Africa AMNH VL 361 | RHS | Bailey et al. (2014) | This work |
| Cova Negra | NEA | Bailey et al. (2014) | This work |
| KDP 1 KRP 45 | NEA | Bailey et al. (2014) | This work |
| KDP 3 | NEA | Bailey et al. (2014) | This work |
| KDP 22 | NEA | Bailey et al. (2014) | This work |
| La Ferrassie 8 | NEA | Bailey et al. (2014) | This work |
| La Quina H18 | NEA | Bailey et al. (2014) | This work |
| Roc de Marsal | NEA | Bailey et al. (2014) | This work |
| Lagar Velho | UPHS | Bailey et al. (2014) | This work |
| Madeleine | UPHS | Bailey et al. (2014) | This work |
| Pataud | UPHS | Bailey et al. (2014) | This work |
| St Germain B6 | UPHS | Bailey et al. (2014) | This work |
| St Germain B7 | UPHS | Bailey et al. (2014) | This work |
| Sunghir 3 | UPHS | Bailey et al. (2014) | This work |
| Veyrier 1 | UPHS | Bailey et al. (2014) | This work |
| Kostenki 15 | UPHS | Bailey et al. (2014) | This work |
| Qafzeh 10 | EHS | Bailey et al. (2014) | This work |
| Qafzeh 15 | EHS | Bailey et al. (2014) | This work |
| Skhul 1 | EHS | Bailey et al. (2014) | This work |
| Dar es Soltan 1 | EHS | Bailey et al. (2014) | This work |
| Dar es Soltan 3H9 | EHS | Bailey et al. (2014) | This work |
| Dar es Soltan H6 | EHS | Bailey et al. (2014) | This work |
| Temara H7 | EHS | Bailey et al. (2014) | This work |
| Fontechevade | UPHS | Bailey et al. (2014) | This work |
| Fossellone | UPHS | Bailey et al. (2014) | This work |
| Goughs Cave | UPHS | Bailey et al. (2014) | This work |
| Laugerie Basse | UPHS | Bailey et al. (2014) | This work |
| Les Rois 19 | UPHS | Bailey et al. (2014) | This work |
| Les Rois | UPHS | Bailey et al. (2014) | This work |
| Mladec 1 | UPHS | Bailey et al. (2014) | This work |
| Mladec 2 | UPHS | Bailey et al. (2014) | This work |
| Pesko | UPHS | Bailey et al. (2014) | This work |
| Sunghir 2 | UPHS | Bailey et al. (2014) | This work |
| St Germain 2 | UPHS | Bailey et al. (2014) | This work |
| La Fate XIII | NEA | Bailey et al. (2014) | This work |
| St Cesaire | NEA | Bailey et al. (2014) | This work |
| Arcy sur Cure 39 | NEA | Bailey et al. (2014) | This work |
| Petit Puymoyen | NEA | Bailey et al. (2014) | This work |
| Monsempron 1953-1 | NEA | Bailey et al. (2014) | This work |
| Le Moustier | NEA | Bailey et al. (2014) | This work |
| Obi Rakhmat | NEA | Bailey et al. (2014) | This work |
| KRP D101 | NEA | Bailey et al. (2014) | This work |
| KRP D171 | NEA | Bailey et al. (2014) | This work |
| KRP maxilla C | NEA | Bailey et al. (2014) | This work |
| KRP maxilla D | NEA | Bailey et al. (2014) | This work |
| GP1 on overall mean | - | This work | This work |
| GP1 on NEA mean | - | This work | This work |
| GP1 on EHS mean | - | This work | This work |
| GP1 on UPHS mean | - | This work | This work |
| GP1 on RHS mean | - | This work | This work |

Table S3

List of the individuals used as comparative sample for 3D GM EDJ and CEJ analysis of GP1.

| Specimen | Side | Comparative group | Source of the 3D models | Source of the coordinates |
| --- | --- | --- | --- | --- |
| BD 8 | L | NEA | Davies et al. (2024) | Davies et al. (2024) |
| Combe Grenal IX^B^ | R | NEA | Davies et al. (2024) | Davies et al. (2024) |
| Combe Grenal XIII | R | NEA | Davies et al. (2024) | Davies et al. (2024) |
| KRP 46 | L | NEA | Davies et al. (2024) | Davies et al. (2024) |
| KRP 47 | L | NEA | Davies et al. (2024) | Davies et al. (2024) |
| KRP 48 | L | NEA | Davies et al. (2024) | Davies et al. (2024) |
| KRP 100 | L | NEA | Davies et al. (2024) | Davies et al. (2024) |
| KRP 174 | R | NEA | Davies et al. (2024) | Davies et al. (2024) |
| La Quina H18 | L | NEA | Davies et al. (2024) | Davies et al. (2024) |
| Roc de Marsal | L | NEA | Davies et al. (2024) | Davies et al. (2024) |
| SCLA 4A 4 | R | NEA | Davies et al. (2024) | Davies et al. (2024) |
| SD 531 | R | NEA | Davies et al. (2024) | Davies et al. (2024) |
| SD 1105 | R | NEA | Davies et al. (2024) | Davies et al. (2024) |
| Gibraltar 2 | R | NEA | ESRF Paleontology database (Smith et al., 2010) | This work |
| KRP 101 | L | NEA | NESPOS | This work |
| KRP 134 | R | NEA | NESPOS | This work |
| KRP 136 on NEA mean | L | NEA | NESPOS | This work |
| KRP 164 on NEA mean | L | NEA | NESPOS | This work |
| KRP 167 on NEA mean | R | NEA | NESPOS | This work |
| Qafzeh 15 | L | EHS | ESRF Paleontology database (Smith et al., 2010) | This work |
| Qafzeh 10 on HS mean | R | EHS | ESRF Paleontology database (Smith et al., 2010) | This work |
| Belgian 59b | L | RHS | Davies et al. (2024) | Davies et al. (2024) |
| Belgian A33 | R | RHS | Davies et al. (2024) | Davies et al. (2024) |
| Belgian A34 | R | RHS | Davies et al. (2024) | Davies et al. (2024) |
| Belgian A37 | L | RHS | Davies et al. (2024) | Davies et al. (2024) |
| Belgian M1D | R | RHS | Davies et al. (2024) | Davies et al. (2024) |
| M 13 | L | RHS | Davies et al. (2024) | Davies et al. (2024) |
| M 51 | R | RHS | Davies et al. (2024) | Davies et al. (2024) |
| M 106 | L | RHS | Davies et al. (2024) | Davies et al. (2024) |
| M 183 | L | RHS | Davies et al. (2024) | Davies et al. (2024) |
| M 186 | R | RHS | Davies et al. (2024) | Davies et al. (2024) |
| MIR 202 N39 24 | L | RHS | Morphosource (Gamarra et al., 2021) | This work |
| MIR 203 S36 151 | R | RHS | Morphosource (Gamarra et al., 2021) | This work |
| MIR 202 T35 M7 | L | RHS | Morphosource (Gamarra et al., 2021) | This work |
| MIR 201 162 | R | RHS | Morphosource (Gamarra et al., 2021) | This work |
| MIR 201 164 | R | RHS | Morphosource (Gamarra et al., 2021) | This work |
| MIR 201 501 | L | RHS | Morphosource (Gamarra et al., 2021) | This work |
| GC 209 | L | RHS | Morphosource (Gamarra et al., 2021) | This work |
| 02 CS 9114 | L | RHS | Morphosource (Gamarra et al., 2021) | This work |
| 02 CS 962 | L | RHS | Morphosource (Gamarra et al., 2021) | This work |
| A 04 12 75 | R | RHS | Morphosource (Gamarra et al., 2021) | This work |
| 02 CS 9348 | L | RHS | Morphosource (Gamarra et al., 2021) | This work |
| GN 95 Rext4 | R | RHS | Morphosource (Gamarra et al., 2021) | This work |
| GN 95 Rext7 | R | RHS | Morphosource (Gamarra et al., 2021) | This work |
| GN M1 1 | L | RHS | Morphosource (Gamarra et al., 2021) | This work |
| GP1 on overall mean | L | - | This work | This work |
| GP1 on NEA mean | L | - | This work | This work |
| GP1 on EHS mean | L | - | This work | This work |
| GP1 on RHS mean | L | - | This work | This work |

| Table S4. Data to perform the correlation analysis of the Neanderthal crown outlines with chronological age and geographic location. | | | | | | |
| --- | --- | --- | --- | --- | --- | --- |
|  |  |  |  |  |  |  |
| Specimen | Country |  | Latitude, Longitude | Chronology | Chronological group for Shapiro-Wilk normality test^‡^ | |
| Cova Negra | Spain | "SP" | 38.9833, -0.5167 ^a^ | 273 ± 26 - 146 ± 34 ka (MIS 8-6) ^e^ | early Neanderthals ^s^ | MIS 8-5 |
| KDP 1 KRP 45 | Croatia | "CR" | 46.15, 15.86 ^a^ | 130 ka (MIS 5e) ^f^ | early Neanderthals ^s^ | MIS 8-5 |
| KDP 3 | Croatia | "CR" | 46.15, 15.86 ^a^ | 130 ka (MIS 5e) ^f^ | early Neanderthals ^s^ | MIS 8-5 |
| KDP 22 | Croatia | "CR" | 46.15, 15.86 ^a^ | 130 ka (MIS 5e) ^f^ | early Neanderthals ^s^ | MIS 8-5 |
| La Ferrassie 8 | France | "FR" | 44.9519, 0.9381 ^a^ | 41.7 - 40.8 ka cal BP (MIS 3) ^g^ | classic Neanderthals ^s^ | MIS 4-3 |
| La Quina H18 | France | "FR" | 45.5072, 0.2928 ^a^ | MIS 4-3 ^h^ | classic Neanderthals ^s^ | MIS 4-3 |
| Roc de Marsal | France | "FR" | 44.8978, 0.9554 ^b^ | 60 - 70 ka (MIS 4) ^i^ | classic Neanderthals ^s^ | MIS 4-3 |
| La Fate XIII | Italy | "IT" | 44.1958, 8.3675 ^a^ | 78 ± 9 ka and 78 ± 13 ka (MIS 5a) ^j^ | early Neanderthals ^s^ | MIS 8-5 |
| St Cesaire | France | "FR" | 45.7489, -0.5053 ^a^ | 36.3 ± 2.7 ka cal BP (MIS 3) ^k^ | classic Neanderthals ^s^ | MIS 4-3 |
| Arcy sur Cure 39 | France | "FR" | 47.5909, 3.7625 ^c^ | 38 ka - 33 ka (MIS 3) ^l^ | classic Neanderthals ^s^ | MIS 4-3 |
| Petit Puymoyen | France | "FR" | 45.6167, 0.1833 ^a^ | MIS 4 ^m^ | classic Neanderthals ^s^ | MIS 4-3 |
| Monsempron 1953-1 | France | "FR" | 44.5046, 0.9294 ^†^ ^a^ | MIS 5 ^n,o^ | early Neanderthals ^s^ | MIS 8-5 |
| Le Moustier | France | "FR" | 44.9950, 1.0656 ^a^ | 56 - 40 ka (MIS 3) ^p^ | classic Neanderthals ^s^ | MIS 4-3 |
| Obi Rakhmat | Uzbekistan | "UZ" | 41.5691, 70.1334 ^d^ | 70 ka (MIS 4) ^q,r^ | classic Neanderthals ^s^ | MIS 4-3 |
| KRP D101 | Croatia | "CR" | 46.15, 15.86 ^a^ | 130 ka (MIS 5e) ^f^ | early Neanderthals ^s^ | MIS 8-5 |
| KRP D171 | Croatia | "CR" | 46.15, 15.86 ^a^ | 130 ka (MIS 5e) ^f^ | early Neanderthals ^s^ | MIS 8-5 |
| KRP maxilla C | Croatia | "CR" | 46.15, 15.86 ^a^ | 130 ka (MIS 5e) ^f^ | early Neanderthals ^s^ | MIS 8-5 |
| KRP maxilla D | Croatia | "CR" | 46.15, 15.86 ^a^ | 130 ka (MIS 5e) ^f^ | early Neanderthals ^s^ | MIS 8-5 |
| GP1 | Italy | "IT" | 40.0003, 15.3820 ^a^ | MIS 6 | early Neanderthals ^s^ | MIS 8-5 |
|  |  |  |  |  |  |  |
| ^a^ Janković et al., 2024. |  |  |  |  |  |  |
| ^b^ Aldeias et al., 2012; the coordinates have been extracted from Google Maps (https://www.google.com/maps). | | | | |  |  |
| ^c^ Gicqueau et al,. 2023; the coordinates have been extracted from Google Maps (https://www.google.com/maps). | | | | |  |  |
| ^†^ The exact coordinates of the site are unknown^a^. We extracted the coordinates of the village Montsempron-Libos, (Lot-et-Garonne, France) from Google Maps (https://www.google.com/maps). | | | | | |  |
| ^d^ Krivoshapkin et al., 2010; the coordinates have been extracted from Google Maps (https://www.google.com/maps). | | | | |  |  |
| ^‡^ The labels "early Neanderthals" and "classic Neanderthals", here used as a grouping strategy for the Shapiro-Wilk test (see Methods section in the main text), should be interpreted according to the definition shared in Profico et al. (2023) ^s^ : early Neanderthals= MIS 7-5; classic Neanderthals= MIS 4-3. | | | | | |  |
|  |  |  |  |  |  |  |
| ^e^ Richard et al., 2019 |  |  |  |  |  |  |
| ^f^ Radovčić et al., 2016 |  |  |  |  |  |  |
| ^g^ Balzeau et al., 2020 |  |  |  |  |  |  |
| ^h^ Frouin et al., 2017 |  |  |  |  |  |  |
| ^i^ Guérin et al., 2012 |  |  |  |  |  |  |
| ^j^ Echassoux et al., 1989 |  |  |  |  |  |  |
| ^k^ Mercier et al., 1991 |  |  |  |  |  |  |
| ^l^ Bailey and Hublin, 2006 |  |  |  |  |  |  |
| ^m^ Ready and Morin, 2019 |  |  |  |  |  |  |
| ^n^ Vallois, 1952 |  |  |  |  |  |  |
| ^o^ El Zaatari et al., 2011 |  |  |  |  |  |  |
| ^p^ Valladas et al., 1986 |  |  |  |  |  |  |
| ^q^ Blackwell et al., 2006 |  |  |  |  |  |  |
| ^r^ Bailey et al., 2008 |  |  |  |  |  |  |
| ^s^ Profico et al., 2023 |  |  |  |  |  |  |

| Table S5. Data to perform the correlation analysis of the Neanderthal EDJs and CEJs with chronological age and geographic location. | | | | | |
| --- | --- | --- | --- | --- | --- |
|  |  |  |  |  |  |
| Specimen | Country | Latitude, Longitude | Chronology | Chronological group for Shapiro-Wilk normality test^‡^ |  |
| BD 8 | France | 45.6706, 0.4461 ^a^ | 151 ka ± 15 ka -101 ka ± 12 ka (MIS 6-5d) ^f^ | early Neanderthals ^k^ | MIS 6-5 |
| Combe Grenal IX^B^ | France | 44.8064, 1.2259 ^a^ | 68 ka - 60 ka (MIS 4) ^g^ | classic Neanderthals ^k^ | MIS 4-3 |
| Combe Grenal XIII | France | 44.8064, 1.2259 ^a^ | 68 ka - 60 ka (MIS 4) ^g^ | classic Neanderthals ^k^ | MIS 4-3 |
| KRP 46 | Croatia | 46.15, 15.86 ^a^ | 130 ka (MIS 5e) ^h^ | early Neanderthals ^k^ | MIS 6-5 |
| KRP 47 | Croatia | 46.15, 15.86 ^a^ | 130 ka (MIS 5e) ^h^ | early Neanderthals ^k^ | MIS 6-5 |
| KRP 48 | Croatia | 46.15, 15.86 ^a^ | 130 ka (MIS 5e) ^h^ | early Neanderthals ^k^ | MIS 6-5 |
| KRP 100 | Croatia | 46.15, 15.86 ^a^ | 130 ka (MIS 5e) ^h^ | early Neanderthals ^k^ | MIS 6-5 |
| KRP 174 | Croatia | 46.15, 15.86 ^a^ | 130 ka (MIS 5e) ^h^ | early Neanderthals ^k^ | MIS 6-5 |
| La Quina H18 | France | 45.5072, 0.2928 ^a^ | MIS 4-3 ^i^ | classic Neanderthals ^k^ | MIS 4-3 |
| Roc de Marsal | France | 44.8978, 0.9554 ^b^ | 60 - 70 ka (MIS 4) ^j^ | classic Neanderthals ^k^ | MIS 4-3 |
| SCLA 4A 4 | Belgium | 50.5105, 5.0236 ^c^ | MIS 5d-5b ^c^ | early Neanderthals ^k^ | MIS 6-5 |
| SD 531 | Spain | 43.3835, -5.3231 ^d^ | 49 ka BP (MIS 3) ^d^ | classic Neanderthals ^k^ | MIS 4-3 |
| SD 1105 | Spain | 43.3835, -5.3231 ^d^ | 49 ka BP (MIS 3) ^d^ | classic Neanderthals ^k^ | MIS 4-3 |
| Gibraltar 2 | Gibraltar | 36.1456, -5.3425 ^e^ | 50 ka - 30 ka (MIS 3) ^e^ | classic Neanderthals ^k^ | MIS 4-3 |
| KRP 101 | Croatia | 46.15, 15.86 ^a^ | 130 ka (MIS 5e) ^h^ | early Neanderthals ^k^ | MIS 6-5 |
| KRP 134 | Croatia | 46.15, 15.86 ^a^ | 130 ka (MIS 5e) ^h^ | early Neanderthals ^k^ | MIS 6-5 |
| KRP 136 on NEA mean | Croatia | 46.15, 15.86 ^a^ | 130 ka (MIS 5e) ^h^ | early Neanderthals ^k^ | MIS 6-5 |
| KRP 164 on NEA mean | Croatia | 46.15, 15.86 ^a^ | 130 ka (MIS 5e) ^h^ | early Neanderthals ^k^ | MIS 6-5 |
| KRP 167 on NEA mean | Croatia | 46.15, 15.86 ^a^ | 130 ka (MIS 5e) ^h^ | early Neanderthals ^k^ | MIS 6-5 |
| GP1 | Italy | 40.0003, 15.3820 ^a^ | MIS 6 | early Neanderthals ^s^ | MIS 6-5 |
|  |  |  |  |  |  |
| ^a^ Janković et al., 2024. |  |  |  |  |  |
| ^b^ Aldeias et al., 2012; the coordinates have been extracted from Google Maps (https://www.google.com/maps). | | | |  |  |
| ^c^ Abrams et al., 2013; the coordinates have been extracted from Google Maps (https://www.google.com/maps). | | | |  |  |
| ^d^ Estalrrich et al., 2017; the coordinates have been extracted from Google Maps (https://www.google.com/maps). | | | |  |  |
| ^e^ Smith et al., 2010; the coordinates have been extracted from Google Maps (https://www.google.com/maps). | | | |  |  |
| ^f^ Blackwell et al., 1992 |  |  |  |  |  |
| ^g^ Mellars, 1986 |  |  |  |  |  |
| ^h^ Radovčić et al., 2016 |  |  |  |  |  |
| ^i^ Frouin et al., 2017 |  |  |  |  |  |
| ^j^ Guérin et al., 2012 |  |  |  |  |  |
| ^‡^ The labels "early Neanderthals" and "classic Neanderthals", here used as a grouping strategy for the Shapiro-Wilk test (see Methods section in the main text), should be interpreted according to the definition shared in Profico et al. (2023) ^k^ : early Neanderthals= MIS 7-5; classic Neanderthals= MIS 4-3. | | | | |  |
|  |  |  |  |  |  |
| ^k^ Profico et al., 2023 |  |  |  |  |  |

| Table S6 |  |  |  |
| --- | --- | --- | --- |
| Results for permutation test for GP1 crown outline | | | |
|  |  |  |  |
|  | EHS | RHS | NEA |
| RHS | 0.16 |  |  |
| NEA | **0.001*** | **0.0006*** |  |
| UPHS | 0.11 | 0.14 | **0.0006*** |
| * Significant differences (p <0.05) | | | |

| Table S7 |  |  |  |  |  |  |  |  |  |  |  |  |
| --- | --- | --- | --- | --- | --- | --- | --- | --- | --- | --- | --- | --- |
| Results of QDA and posterior probability for GP1 crown outline | | | | | | | | | | | |  |
|  |  |  |  |  |  |  |  |  |  |  |  |  |
|  |  | EHS+UPHS+RHS |  | NEA |  |  |  |  | EHS+UPHS+RHS |  | NEA |  |
| Tooth class | PCs | Correct % |  | Correct % |  | Total accuracy |  | Specimen | P post % |  | P post % |  |
|  |  |  |  |  |  |  |  |  |  |  |  |  |
| UM1 | PC 1-3 | 95.3 |  | 94.4 |  | 95.2 |  | GP1 | 4.38 |  | 95.6 |  |
|  |  |  |  |  |  |  |  |  |  |  |  |  |

| Table S8  Results of Pearson’s product-moment correlation for GP1 crown outline | | | |
| --- | --- | --- | --- |
|  | PC1 | PC2 | PC3 |
|  |  |  |  |
| Latitude | 0.437 | 0.355 | 0.539 |
| Longitude | 0.101 | 0.736 | 0.378 |
| * Significant differences (p <0.05) | | | |

| Table S9 |  |  |  |  |
| --- | --- | --- | --- | --- |
| Results of permutation test for GP1 EDJ and CEJ | | | |  |
|  |  |  |  |  |
|  | EHS | NEA |  |  |
| NEA | 0.47 |  |  |  |
| RHS | 0.47 | **0.0003*** |  |  |
| * Significant differences (p <0.05) | | |  |  |

| Table S10 |  |  |  |  |  |  |  |  |  |  |  |  |
| --- | --- | --- | --- | --- | --- | --- | --- | --- | --- | --- | --- | --- |
| Results of LDA and posterior probability for GP1 EDJ and CEJ | | | | |  |  |  |  |  |  |  |  |
|  |  |  |  |  |  |  |  |  |  |  |  |  |
|  |  | EHS+RHS |  | NEA |  |  |  |  | EHS+RHS |  | NEA |  |
| Tooth class | PCs | Correct % |  | Correct % |  | Total accuracy |  | Specimen | P post % |  | P post % |  |
|  |  |  |  |  |  |  |  |  |  |  |  |  |
| UM1 | PC 1-7 | 100 |  | 100 |  | 100 |  | GP1 | 0 |  | 100 |  |
|  |  |  |  |  |  |  |  |  |  |  |  |  |

Table S11

| Results of Pearson’s product-moment correlation for GP1 EDJ and CEJ | | | |
| --- | --- | --- | --- |
|  | PC1 | PC2 | PC3 |
|  |  |  |  |
| Latitude | 0.273 | 0.662 | 0.760 |
| Longitude | **0.0004*** | **0.032*** | 0.059 |
| * Significant differences (p <0.05) | | | |

REFERENCES

Abrams, G., S. M. Bello, K. Di Modica, S. Pirson, and D. Bonjean. 2014. “When Neanderthals Used Cave Bear (Ursus Spelaeus) Remains: Bone Retouchers From Unit 5 of Scladina Cave (Belgium).” *Quaternary International* 326: 274–287. <https://doi.org/10.1016/j.quaint.2013.10.022>.

Aldeias, V., P. Goldberg, D. Sandgathe, et al. 2012. “Evidence for Neandertal Use of Fire at Roc de Marsal (France).” *Journal of Archaeological Science* 39, no. 7: 2414–2423. <https://doi.org/10.1016/j.jas.2012.01.039>.

Bailey, S., M. Glantz, T. D. Weaver, and B. Viola. 2008. “The Affinity of the Dental Remains From Obi‐Rakhmat Grotto, Uzbekistan.” *Journal of Human Evolution* 55, no. 2: 238–248. <https://doi.org/10.1016/j.jhevol.2008.03.004>.

Bailey, S. E., and J. J. Hublin. 2006. “Did Neanderthals Make the Châtelperronian Assemblage From La Grotte du Renne (Arcy‐Sur‐Cure, France)?” In *Neanderthals Revisited: New Approaches and Perspectives*, 191–209. Springer Netherlands.

Balzeau, A., A. Turq, S. Talamo, et al. 2020. “Pluridisciplinary Evidence for Burial for the La Ferrassie 8 Neandertal Child.” *Scientific Reports* 10, no. 1: 21230. <https://doi.org/10.1038/s41598-020-77611-z>.

Blackwell, B., A. Mian, S. Baboumian, et al. 2006. “Settling the Age Dispute for the Late Middle Paleolithic at the Obi‐Rakhmat Hominid Site, Uzbekistan.” Presentation at the 2006 Paleoanthropology Society Meetings.

Blackwell, B., N. Porat, H. P. Schwarcz, and A. Debenath. 1992. “ESR Dating of Tooth Enamel: Comparison With ^230^Th^234^U Speleothem Dates at La Chaise‐de‐Vouthon (Charente), France.” *Quaternary Science Reviews* 11, no. 1–2: 231–244. <https://doi.org/10.1016/0277-3791(92)90068-J>.

Echassoux, A., F. D'Errico, and G. Giacobini. 1989. “Les Nouvelles Fouilles Dans le Gisement Mousterien de la Caverna Delle Fate (Finale, Ligurie Italienne).” In *L'Homme de Néandertal, vol. 6: La Subsistance*, edited by M. Otte, 49–58. ERAUL.

El Zaatari, S., F. E. Grine, P. S. Ungar, and J. J. Hublin. 2011. “Ecogeographic Variation in Neandertal Dietary Habits: Evidence From Occlusal Molar Microwear Texture Analysis.” *Journal of Human Evolution* 61, no. 4: 411–424. <https://doi.org/10.1016/j.jhevol.2011.05.004>.

Estalrrich, A., S. El Zaatari, and A. Rosas. 2017. “Dietary Reconstruction of the El Sidrón Neandertal Familial Group (Spain) in the Context of Other Neandertal and Modern Hunter‐Gatherer Groups. A Molar Microwear Texture Analysis.” *Journal of Human Evolution* 104: 13–22. <https://doi.org/10.1016/j.jhevol.2016.12.003>.

Frayer, D. W. 1978. *Evolution of the Dentition in Upper Paleolithic and Mesolithic Europe*. Vol. 10. University of Kansas Publications in Anthropology.

Frouin, M., C. Lahaye, H. Valladas, et al. 2017. “Dating the Middle Paleolithic Deposits of La Quina Amont (Charente, France) Using Luminescence Methods.” *Journal of Human Evolution* 109: 30–45. <https://doi.org/10.1016/j.jhevol.2017.05.002>.

Gicqueau, A., A. Schuh, J. Henrion, et al. 2023. “Anatomically Modern Human in the Châtelperronian Hominin Collection From the Grotte du Renne (Arcy‐Sur‐Cure, Northeast France).” *Scientific Reports* 13, no. 1: 12682. <https://doi.org/10.1038/s41598-023-39767-2>.

Guérin, G., E. Discamps, C. Lahaye, et al. 2012. “Multi‐Method (TL and OSL), Multi‐Material (Quartz and Flint) Dating of the Mousterian Site of Roc de Marsal (Dordogne, France): Correlating Neanderthal Occupations With the Climatic Variability of MIS 5–3.” *Journal of Archaeological Science* 39, no. 10: 3071–3084. <https://doi.org/10.1016/j.jas.2012.04.047>.

Hillson, S. W., and E. Trinkaus. 2002. “Comparative Dental Crown Metrics.” In *Portrait of the Artist as a Child*, edited by J. Zilhao and E. Trinkaus, 356–364. Instituto Portugues de Arqueologia.

Krivoshapkin, A. I., Y. V. Kuzmin, and A. T. Jull. 2010. “Chronology of the Obi‐Rakhmat Grotto (Uzbekistan): First Results on the Dating and Problems of the Paleolithic Key Site in Central Asia.” *Radiocarbon* 52, no. 2: 549–554. <https://doi.org/10.1017/S0033822200045586>.

Mellars, P. 1986. “A New Chronology for the French Mousterian Period.” *Nature* 322: 410–411. <https://doi.org/10.1038/322410a0>.

Mercier, N., H. Valladas, J. L. Joron, J. L. Reyss, F. Lévèque, and B. Vandermeersch. 1991. “Thermoluminescence Dating of the Late Neanderthal Remains From Saint‐Césaire.” *Nature* 351, no. 6329: 737–739. <https://doi.org/10.1038/351737a0>.

Radovčić, D., D. Japundžić, A. O. Sršen, J. Radovčić, and D. W. Frayer. 2016. “An Interesting Rock From Krapina.” *Comptes Rendus Palevol* 15, no. 8: 988–993. <https://doi.org/10.1016/j.crpv.2016.04.013>.

Ready, E., and E. Morin. 2019. “Preliminary Analysis of Faunal Remains From Three Middle Paleolithic Deposits in Charente, France.” *Journal of Archaeological Science: Reports* 24: 290–301. <https://doi.org/10.1016/j.jasrep.2019.01.020>.

Richard, M., C. Falguères, E. Pons‐Branchu, et al. 2019. “ESR/U‐Series Chronology of Early Neanderthal Occupations at Cova Negra (Valencia, Spain).” *Quaternary Geochronology* 49: 283–290. <https://doi.org/10.1016/j.quageo.2018.05.004>.

Shpakova, E. G. 2001. “Palaeolithic Human Dental Remains From Siberia.” *Archaeology, Ethnology and Anthropology of Eurasia* 4: 64–76.

Suzuki, H., and F. Takai. 1970. *The Amud Man and His Cave Site*. Academic Press of Japan.

Teschler‐Nicola, M. 2006. *Early Modern Humans at the Moravian Gate: The Mladec Caves and Their Remains*. Springer.

Trinkaus, E. 1983. *The Shanidar Neanderthals*. Academic Press.

Valladas, H., J. M. Geneste, J. L. Joron, and J. P. Chadelle. 1986. “Thermoluminescence Dating of Le Moustier (Dordogne, France).” *Nature* 322, no. 6078: 452–454.

Vallois, H. V. 1952. “Les Restes Humains du Gisement Moustérien de Monsempron.” *Annales Paléorient* 38: 100–120.

Vandermeersch, B. 1981. *Les Hommes Fossiles de Qafzeh*. Éditions du Centre National de la Recherche Scientifique.

Wolpoff, M. H. 1979. “The Krapina Dental Remains.” *American Journal of Physical Anthropology* 50: 67–114. <https://doi.org/10.1002/ajpa.1330500110>.
